# Immune priming modulates *Galleria mellonella* and *Pseudomonas entomophila* interaction. Antimicrobial properties of Kazal peptide Pr13a

**DOI:** 10.3389/fimmu.2024.1358247

**Published:** 2024-02-26

**Authors:** Michał Sułek, Jakub Kordaczuk, Paweł Mak, Justyna Śmiałek-Bartyzel, Monika Hułas-Stasiak, Iwona Wojda

**Affiliations:** ^1^ Department of Immunobiology, Institute of Biological Sciences, Maria Curie-Sklodowska University, Lublin, Poland; ^2^ Department of Analytical Biochemistry, Faculty of Biochemistry, Biophysics and Biotechnology, Jagiellonian University, Kraków, Poland; ^3^ Doctoral School of Exact and Natural Sciences, Jagiellonian University, Kraków, Poland; ^4^ Department of Functional Anatomy and Cytobiology, Institute of Biological Sciences, Maria Curie-Sklodowska University, Lublin, Poland

**Keywords:** Galleria mellonella, Pseudomonas entomophila, immune priming, antimicrobial peptides, Kazal peptide Pr13a

## Abstract

*Galleria mellonella* larvae repeatedly infected with *Pseudomonas entomophila* bacteria re-induced their immune response. Its parameters, i.e. the defence activities of cell-free hemolymph, the presence and activity of antimicrobial peptides, and the expression of immune-relevant genes were modulated after the re-challenge in comparison to non-primed infected larvae, resulting in better protection. No enhanced resistance was observed when the larvae were initially infected with other microorganisms, and larvae pre-infected with *P. entomophila* were not more resistant to further infection with other pathogens. Then, the peptide profiles of hemolymph from primed- and non-primed larvae infected with *P. entomophila* were compared by quantitative RP-HPLC (Reverse Phase - High Performance Liquid Chromatography). The level of carbonic anhydrase, anionic peptide-1, proline peptide-2, and finally, unknown so far, putative Kazal peptide Pr13a was higher in the primed infected animals than in the larvae infected with *P. entomophila* for the first time. The expression of the Pr13a gene increased two-fold after the infection, but only in the primed animals. To check whether the enhanced level of Pr13a could have physiological significance, the peptide was purified to homogeneity and checked for its defence properties. In fact, it had antibacterial activity: at the concentration of 15 µM and 7.5 µM it reduced the number of *P. entomophila* and *Bacillus thuringiensis* CFU, respectively, to about 40%. The antibacterial activity of Pr13a was correlated with changes observed on the surface of the peptide-treated bacteria, e.g. surface roughness and adhesion force. The presented results bring us closer to finding hemolymph constituents responsible for the effect of priming on the immune response in re-infected insects.

## 1 Introduction

Larvae of the greater wax moth *Galleria mellonella* inhabit honeybee hives or, more often, earwax slices after wax centrifugation stored to be melted into wax. The defence abilities of this insect against microbial infections have been intensively studied for several years, since they show some common aspects with the innate immunity of vertebrates (1). Haemocytes circulating in *G. mellonella* hemolymph with functional similarities to human neutrophils phagocytose intruders directly after they are recognised. Groups of microorganisms or parasites can be entrapped in multihemocyte structures called nodules or capsules, respectively (2–5). Quite recently, formation of extracellular traps by *G. mellonella* haemocytes contributing to immobilisation and finally killing of microbes has been described (6). Soluble effector molecules contribute to the humoral immune response. It includes phenol oxidase (PO) released from oenocytoides after infection, leading to melanin synthesis and antimicrobial activity (3, 7). Independently, about 20 antimicrobial peptides have been found in *G. mellonella*. These include cecropin A and D, gallerimycin, galiomycin, defensin-like peptide, anionic peptide 1 and 2, proline-rich peptides 1 and 2, gloverins, moricins, heliocin-like peptide, and GmCP8 (8–14). Most of them are up-regulated after infection due to the activation of homologues of Toll and Imd pathways, which were first found in *Drosophila melanogaster* (15–17). In the hemolymph, peptide components cooperate with each other to provide maximum protection against infecting microbes. For instance, lysozyme acts synergistically with cecropin and anionic peptide- 2, while cecropin with gallerimycin act against Gram-negative bacteria (18, 19).

Recent investigations point to the great adaptive abilities of the insect immune system. Its reaction to infection can be affected by both abiotic and biotic external conditions. Abiotic conditions include ambient temperature, humidity, and access to food, while biotic ones include the presence of predators and previous immune experience (20). The last one refers to the previous contact with a pathogen, which gives enhanced resistance in the case of repeated infection. The first reports concerning increased resistance of *G. mellonella* pre-injected with attenuated bacteria against normally lethal doses of microorganism appeared as early as in the 1920’s and 1930’s (21, 22), followed by further reports a few decades later (23–25). However, recent years have brought more interest in this phenomenon. In 2004, the occurrence of increased immunity after previous contact with a pathogen was named immunological or immune priming (26). At the outset, greater attention was directed towards exposing individuals to lipopolysaccharides (LPS) along with other components of microbial cell walls, deceased microorganisms, and non-pathogenic organisms. This was followed by testing survival curves subsequent to the exposure to pathogenic microorganisms. There are reports that protection lasted as long as the immune parameters induced after the first challenge were up-regulated (examples: 27, 28). Moreover, there are reports that an infection with a given pathogen may protect against not only the same but also other pathogens, which indicates that the response can be non-specific, probably because of the up-regulation of general immune parameters. At present, this phenomenon is rather called immune enhancement or immune protection (29). Nowadays, immune priming is understood as an analogue of the immune memory of vertebrate animals. Real “memorisation” of previous infection can be considered when the above-mentioned immune parameters induced after the first challenge go back to the initial level or are stabilised; then, the defence response is re-induced in response to the second immune challenge. Another possibility is that the immune response to the initial infection and the re-infection has differently distributed accents. For example, the cellular branch in the first case and humoral mechanisms in the second case may dominate (30–33). Priming resembles vertebrate immune memory when it shows specificity, i.e. increased resistance is targeted against the same pathogen that had primed the immune system of insects (34–38). Finally, the better protection in re-infection lasts longer than in the case of immune enhancement, which also resembles the real immune memory. Such long-lasting specific priming was shown in cockroaches (39), *Bombus terrestris* (40), and *G*. *mellonella* (41–43). The specificity discriminating even bacterial strain was reported in the red flour beetle *Tribolium castaneum* (44). It is important to mention here that, unlike vertebrate immune memory, insect immune priming is not a rule. Probably insects “invest” in priming only when there is a high risk of encountering the same pathogen for the second time, for example, when an insect inhabits the same ecological niche constantly, as their pathogens do. Then, it is “worth” investing in priming than suffering from constant infections (45).

Full detailed mechanisms explaining the immune priming phenomenon are unknown to date. One of the possibilities is the diversity of Dscam receptors, based on alternative splicing, which may provide more efficient recognition in re-infected insects (46). Notwithstanding, no direct evidence has been provided so far. Recent research has paid attention to epigenetic changes induced by pathogens in their infected host, such as methylation or acetylation of DNA, resulting in changes in chromatin condensation followed by changes in gene expression. Such changes may be transduced to the progeny, creating trans-generational immune priming (47–49). Extensive studies are required to find some common points in the mechanisms of the immune priming phenomenon in different host-pathogen models.

Here we present the results of immune priming of *G. mellonella* with entomopathogenic bacteria *Pseudomonas entomophila.* The strain used in the study was isolated from the gut of a *D. melanogaster* female by F. Boccard’s team in Guadeloupe in 2005 (50, 51) and shown to infect the insect *via* the oral route. *P. entomophila* is a Gram-negative bacterium with one polar cilium; it is able to grow in a temperature range from 4 °C to 42 °C with the optimum at 30 °C and pH 5-9 (52, 53). Its genome was sequenced in 2006 and it appeared that the bacterium has a wide set of genes whose products are characterised by high pathogenicity to insects (51). It contains genes encoding such virulence factors as monalysin, protease AprA, and endolysin (54–56). Monalysin belongs to pore-forming toxins responsible for gut perforation (57). It can be activated in the gut by AprA protease, which also promotes gut destruction in the infected host and can digest its immune effectors (55, 57). In turn, endolysin is a cyclic lipopeptide with haemolytic properties; however, its role in *P. entomophila* virulence is not fully known (58, 59). The other *P. entomophila* genes encode toxins TccC and TccD, but their role in pathogenicity is not known (51, 52).

To investigate the immune response of *G. mellonella* to repeated infection with *P. entomophila*, we chose injection as the infection route, which mimics infection through injury happening in nature, since *G. mellonella* shows opportunistic cannibalism (60). Also, injection allows establishing the exact time 0 of infection and ensures that exactly the same number of pathogens enter the hemocel at the same time, which is not possible in the oral route of infection. Also, injection gives the possibility to analyse the systemic immune response, avoiding increased resistance of anatomic barriers to the pathogen attack. We showed that *G. mellonella* larvae pre-exposed to a very low number of bacteria had a higher survival rate, and certain immune parameters were enhanced upon the secondary infection with the same bacteria. We compared the profiles of low-molecular components in primed and non-primed individuals and identified those showing quantitative differences. Among them, there were polypeptides with known function in insect immunity, but we also identified a new polypeptide, i.e. putative (so far) Kazal peptide Pr13a, whose amount was up-regulated in the primed infected group. This polypeptide was purified and characterised for its antimicrobial properties.

## 2 Materials and methods

### 2.1 Insects, microorganisms, and infection

*G. mellonella* (Lepidoptera: Pyralidae) larvae were reared on honeybee nest debris at 28 °C and 60% humidity in darkness. Last instar larvae were used for infection with the *P. entomophila* L48 strain. The bacteria were grown overnight in Luria Broth (LB) medium at 30 °C with shaking, then centrifuged 8 500 ×g and suspended to the density of 10, 50, and 500 cells in 5 µl of PBS (Phosphate Buffered Saline; 140 mM NaCl, 2.68 mM KCl, 10 mM Na_2_HPO_4_, 1.76 mM KH_2_PO_4_, pH 7.4). The number of cells was estimated with the use of a cell counter (Muse Cell Analyzer, MERCK Millipore), followed by plating the cells on 2% agar LB medium and calculation of grown CFU (Colony Forming Units). The larvae were injected with the use of a Hamilton syringe in the last or penultimate proleg previously sterilised with 70% ethanol. Then, they were placed on sterile filter paper in well-ventilated plastic boxes with access to food and kept at 28°C for the indicated time.

The double infection of *G. mellonella* larvae to check the priming effect was done in the following way: one group of larvae was pre-treated with a low dose of *P. entomophila* (10 cells per larva) or other bacteria suspended in PBS, while the comparative group received PBS buffer alone. The larvae were reared for 72 h at 28 °C with access to food. Then, the same *G. mellonella* larvae were infected with the high (500 cells) dose of *P. entomophila* or other microorganisms, as shown in **Table 1**.

**Table 1:** List of microorganisms, growth conditions, and doses used to immunise *G. mellonella* larvae.

Microorganism (source)	Growth conditions	Dose for pre-treatment (priming)	Dose for infection
*Pseudomonas entomophila* L48 F. Boccard, CNRS, Gif-sur-Yvette, France	LB 30 °C	10 cells/5µl/larva	5×10^2^ cells/5µl/larva
*Pseudomonas aeruginosa* ATCC 27853	LB 37 °C	2×10^5^ heat-killed cells/5µl/larva	4×10^4^cells/5µl/larva
*Bacillus thuringiensis* subsp. *kurstaki* HD1 Bacillus Genetic Stock Centre, The Ohio State University, Department of Biochemistry	LB 37 °C	6×10^1^ cells/5µl/larva	6×10^3^cells/5µl/larva
*Candida albicans* ATCC 10231	YPD 37 °C	2× 10^4^ cells/5µl/larva	2×10^5^ cells/5µl/larva

Hemolymph, fat body, or entire animals were collected directly before the second injection (72 h after the first injection) and the respective samples were named NP 0h, P 0h (non-primed, time 0; primed, time 0), and NP and P with the number describing the number of hours after re-injection). The list of microorganisms used for priming and infection is provided in **Table 1**.

For the survival analysis, the infected and control larvae were reared as mentioned above. The number of live animals was counted at the given time points post-infection. The larvae were considered dead when no movement was noticed after touching. The results are presented with the use of the Kaplan-Meier estimator (61).

### 2.2 Histology and TUNEL reaction

For histology, live *G. mellonella* larvae were cooled down and fixed in 10% buffered formalin for 7 days at room temperature, dehydrated, and embedded in Paraplast (Sigma-Aldrich, St. Louis, MO, USA). The samples were then serially sectioned at 5-μm thickness on a rotary microtome and mounted on polysine-coated glass slides (Thermo Fisher Scientific, Braunschweig, Germany). Paraplast sections were dewaxed in xylene (2 x 5 min), rehydrated in decreasing concentrations of ethanol (100%, 96%, and 70% for 5, 3, and 3 min, respectively), and stained with Masson- Goldner’s trichrome (62). The slides were then dehydrated for 3 minutes in each ethanol solution (70%, 96%, 100%), cleared in xylene (2 x 5 min), and finally mounted in xylene dibutyl phthalate (DPX, Sigma-Aldrich, St. Louis, MO, USA). All slides were examined using an Axiovert 200M confocal microscope coupled with a digital camera AxioCamHRc and software Axio Vision 4.8 (Carl Zeiss, Jena, Germany).

For identification of apoptotic cells, the TUNEL reaction was performed. Paraffin-embedded sections were deparaffinised in xylene, rehydrated in ethanol, and rinsed with water. Tissue sections were then heated in a pressure cooker (1 minute in 10 mM citrate buffer, pH 6.0) and washed with PBS. TUNEL-positive cells were identified according to the manufacturer’s protocol of the ApopTag^®^ Peroxidase *In Situ* Apoptosis Detection Kit (Chemicon International, Melbourne, Australia). Briefly, endogenous peroxidase activity was blocked by incubating slides with 3% hydrogen peroxide in phosphate-buffered saline for 10 min. Equilibration buffer was then applied directly to the slides for 15 minutes. Digoxigenin-deoxyuridine triphosphate was incorporated with working strength terminal deoxynucleotidyl transferase in a humidified chamber at 37 °C for 1 hour. The reaction was stopped by 10-minute incubation in Stop/Wash buffer. The antidigoxigenin-peroxidase conjugate was then added and incubated for 30 minutes at room temperature. Staining was developed with diaminobenzidine (DAB), and sections were counterstained with Mayer’s haematoxylin. A negative control without the active TdT enzyme was performed. Only cells stained dark brown were interpreted as TUNEL-positive apoptotic cells. The slides were examined and photographed using an LSM5 Pascal microscope (Carl Zeiss, Jena, Germany) connected to a digital camera (AxioCam HRc, Carl Zeiss, Jena, Germany) and a computer.

### 2.3 Collection of hemolymph and preparation of cell-free hemolymph

The larvae were anesthetised by cooling down on ice before collection of hemolymph. The abdominal side of the larva was washed with 70% ethanol and injured with a sterile scalpel. The leaking hemolymph was collected to sterile Eppendorf tubes containing a few crystals of phenylthiourea to prevent melanisation. The tubes were then centrifuged 200 × g at 4 °C to sediment haemocytes, and the supernatants were transferred to new tubes and centrifuged again at 20 000 × g at 4 °C to remove any debris. The clear cell- and debris-free hemolymph was transferred to new tubes and kept at -20 °C until use.

### 2.4 Determination of antibacterial, phenol oxidase, and lysozyme activity in cell-free hemolymph

For determination of antimicrobial activity in every single experiment, the hemolymph from 10 larvae from a given group was pooled to one Eppendorf tube. The antimicrobial properties of the hemolymph were assessed with an inhibition zone assay with the use of LB plates (0.7% w/v agar, 10 ml for anti-*E. coli*, and 8 ml for anti-*P. entomophila* activity) containing a suspension of *E. coli* D31 (CGSC5165, 63) or *P. entomophila* L48 cells. The hemolymph (5 µl containing 200 µg of total protein for anti-*E. coli* activity and 4 µl containing 400 µg of total protein for anti-*P. entomophila* activity) was applied to the holes in the agar and, after overnight incubation at 37 °C (plates with *E. coli*) or 30 °C (for *P. entomophila*), the areas of growth inhibition zones were measured and the results were quantified according to Hultmark (64) with cecropin B used as a standard.

For determination of lysozyme activity, *Micrococcus luteus* cells (ATCC 4698) were manually homogenised and suspended in Sorensen’s buffer (66 µM KH_2_PO4, 66 mM Na_2_HPO_4_, pH 6.4) in the amount of 0.45 mg of lyophilisate per 1 ml of buffer. After boiling, 195 µl portions of the suspension were added to the wells of a 96-well plate, mixing it carefully each time. Then, a 5-µl portion of the hemolymph containing 200 µg of total protein or water (as a blank control) was added to each well. The absorbance at λ=450 nm was measured directly (time 0) with the use of a Benchmark Plus Microplate Reader (Bio-Rad, USA). Clarification of the solution during *M. luteus* peptidoglycan digestion by lysozyme present in the hemolymph was measured at the indicated time points.

To measure the activity of phenol oxidase present in the collected hemolymph, 18 µl of buffer A (50 mM Tris-HCl, 150 mM NaCl, 5mM CaCl_2_; pH 7.4) and 2 µl of hemolymph sample containing 80 µg of total protein or water (blank sample) were added to the wells of a 96-well plate. After 20-min incubation at room temperature, 180 µl of a 2 mM solution of L-DOPA in buffer B (50 mM Na_2_HPO_4_, 50 mM NaH_2_PO_4_; pH 6.5) was added and the absorbance at λ=490 nm, proportional to melanin synthesis, was measured directly at the indicated time points.

### 2.5 Bioautography

For determination of the antibacterial activity of the separated hemolymph polypeptides (100 µg protein portion per gel well), 13.8% polyacrylamide gel was prepared and electrophoresis was performed according to Laemmli (65). After separation, the gel was washed 2 times for 30 min each in 2.5% of Triton X-100, then in 50 mM Tris-HCl, pH 7.5 (2 times for 15 minutes), and finally in LB medium (2 times for 15 minutes). The gel was overlaid with LB containing 0.7% w/v agar and *E. coli* D31 cells (10 µl of overnight culture for 10 ml of medium), supplemented with 5 mg/ml of lysozyme and streptomycin (0.15 mg/ml). After incubation at 37 °C, the growth inhibition zones were measured using an image analyser Chemi Doc MP ImagingSystem (Bio Rad).

### 2.6 Determination of relative gene expression in the fat body of *G. mellonella* larvae

Dissection of fat bodies from *G. mellonella* larvae, RNA isolation, reverse transcription, and sequence of most primers and Real-Time PCR was performed as described before (66). The primers for *Kazal peptide Pr13a* were as follows: forward: *5’-CCTGCCGGCCCGATA-*3’, reverse: 5’*-CAGTGACTTGGCTCCATTCTT-*3’; for *carbonic anhydrase*: forward 5’-*CCAACAGCGCCACCTAGTG*-3’, reverse: 5’-*TGTCGTCGCAGAAGGAACAG*-3’. Shortly, total RNA was isolated with the use of Gen Elute Mammalian Total RNA Extraction Kit (Sigma). For reverse transcription 1 µg of total RNA and random hexamer primers were taken (High Capacity cDNA Reverse Transcription Kit, Life Technology). Quantitative PCR was performed using Step One Plus PCR System (Applied Biosystems) using the following conditions: 95 °C 10 min, 44 × (95 °C, 15 s—denaturation; 60 °C, 1 min—annealing and extension). As a standard curve, PCR amplification was performed with several dilutions of the cDNA template from the mix of immunized and naive larvae. The relative expression was normalised to the values of ribosomal protein S7e mRNA (reference gene), whose level was constant in all the conditions tested. The relative gene expression was calculated taking into account the efficiency of reaction (67, 68). In every single experiment performed, the fat body from 5 larvae in every group was pooled and used for RNA extraction.

### 2.7 Extraction of low-molecular weight hemolymph components for Tris-Tricine electrophoresis

To obtain methanol extracts, the hemolymph was diluted ten times in a mixture containing methanol, water, and acetic acid (90:9:1, v/v). High molecular proteins were sedimented at 20 000 × g for 30 min. The upper fraction was carefully collected and lyophilised. For HPLC, the extracts were resuspended in 0.1% (v/v) trifluoroacetic acid (TFA) in the volume of two thirds of the initial volume (n) of hemolymph (2/3n), and then the same volume of n-hexane was added. After thorough mixing, the samples were centrifuged for 15 minutes at 4 °C. The upper fraction containing lipids was removed and ethyl acetate was added to the bottom part, mixed, and centrifuged again. The lipid-free bottom part was transferred to new Eppendorf tubes and freeze-dried.

Tris-Tricine electrophoresis was performed and the gel was stained as described by Schagger and von Jagow (69). In the case of separation of fractions collected after reversed phase high pressure liquid chromatography (RP-HPLC), the resolved proteins were electroblotted on a PVDF membrane, stained using Coomassie Blue and visualised using Image Lab software (BioRad) as described earlier (70).

### 2.8 RP-HPLC separation of hemolymph and purification of Kazal peptide Pr13a

The hemolymph extracts obtained as described above were dissolved in 0.1% (v/v) TFA and subjected to RP-HPLC using an UltiMate 3000 HPLC apparatus (Thermo, Waltham, MA, USA) and a Discovery Bio Wide Pore C18 4.6 mm × 250 mm column (Sigma-Aldrich, St. Louis, MO, USA). The separations performed to compare the level of polypeptides were carried out at 40 °C using two buffers A: 0.1% TFA (v/v) and B: 0.07% TFA containing 80% acetonitrile (both v/v), a linear gradient from 20 to 67% of buffer B in 45 min, a 1 ml/min flow rate, and spectrophotometric detection at 220 and 280 nm.

To purify Kazal peptide Pr13a, the fraction from the above separation, eluting at 15-17 min, was collected and subjected to re-chromatography using the same column as above and a linear gradient from 27 to 33% of buffer B in 30 min developed at 50 °C. The fraction eluting at 15-17 min was again collected and subjected to final purification using the same column as above and a linear gradient from 21 to 31% of buffer B in 20 min developed at 45 °C. The peak eluting at 14.5 min was collected, evaporated to dryness, and stored at −20 °C until further assays. The homogeneity and identity of the purified Pr13a peptide were confirmed by SDS/PAGE electrophoresis and N-terminal amino acid sequencing performed using an automatic protein sequencer (PPSQ-31A, Shimadzu, Kyoto, Japan). The quantitation of the peptide in the solution was determined by the bicinchoninic acid assay (BCA, Sigma-Aldrich, St. Louis, MO, USA) calibrated by bovine serum albumin.

### 2.9 *In vitro* assay of the antimicrobial activity of Kazal peptide Pr13a

The antibacterial activity of purified Kazal peptide Pr13a was determined as described in Kordaczuk et al. (70). Shortly, bacteria at the logarithmic growth phase were diluted to OD_600_ of 0.02 and 20 µl portion of this suspension was mixed with the studied peptide solution or water as a control. Each sample was immediately divided into two parts, 10 µl each. One pair (bacteria with the polypeptide and one with water) was incubated for 60 min at 30 °C (*P. entomophila*) or 37 °C (*B. thuringiensis*) with shaking and then plated, while the second pair was plated immediately (time 0). The final concentration of the peptide was 7.5 µM or 15 µM. For plating, the samples were diluted 100, 1 000, and 10 000 times with LB (900 µl of each dilution was added to LB containing soft (0.7%, (w/v)) agar cooled down to 40 °C) and poured on Petri plates. The plates were incubated at 30 °C (*P. entomophila*) or 37 °C (*B. thuringiensis*) until colonies were clearly visible (overnight). The results are presented as a percentage of CFU that appeared after plating the polypeptide-containing samples in relation to the respective CFU obtained from samples without the peptide (70).

### 2.10 Testing the inhibitory activity of Kazal peptide Pr13a against selected proteases

The assay of the inhibitory activity of the peptide was based on spectrophotometric measurement of azocasein digestion by proteases in the presence of the tested compound.

Pr13a was incubated with (i) 1 pmol of thermolysin (Sigma-Aldrich) dissolved in acetate buffer (10 mM sodium acetate, 5 mM calcium acetate, pH 7.5), (ii) 6 pmol of trypsin (Sigma-Aldrich) dissolved in the reaction buffer (10 mM Tris-HCl pH 8.0, 0.1 mM HCl), or, (iii) 0.02 units (U) of elastase (Sigma-Aldrich) dissolved in 10 mM Tris HCl pH 8.0.

A sample incubated with water instead of a protein compound was the control, and the total volume of the mixture was 5 μl. After 15 minutes of pre-incubation at room temperature, 5 μl of azocasein suspension in a water (5 mg/ml) was added to the samples and incubated for 60 minutes at 30 °C. The reaction was stopped by adding 10 μl of a 5% trichloroacetic acid (TCA) solution. The samples were then incubated at room temperature for 10 minutes and centrifuged at 14 000×g for 5 min to settle undigested azocasein. 18 μl of the supernatant containing the formed azopeptides was pipetted into the wells of a Corning^®^ 96 Well Half Area Microplate (Corning, USA), and 9 μl of a 5% NaOH solution was added to enhance the colour of the solution. The absorbance was measured at λ = 450 nm against a blank sample containing only solvent instead of protease. The measurement was performed with a Benchmark spectrophotometer Plus Microplate Reader (Bio-Rad, USA).

### 2.11 Atomic force microscopy

Bacterial samples were prepared for atomic force microscopy (AFM) according to the method described earlier (70). Briefly, log-phase *P. entomophila* and *B. thuringiensis* (OD_600 _= 0.02) in LB were cultivated for 1 h at 30 °C or 37 °C, respectively, with Kazal peptide Pr13a or with water (control) in the total volume of 300 µl. Then, 300 µl of 20 mM phosphate buffer, pH 6.8, was added. Next, the pellets were gently washed twice with 20 mM phosphate buffer, pH 6.8, and twice with non-pyrogenic water. After final centrifugation, the microorganisms were suspended in 10 µl of non-pyrogenic water, applied onto the surface of freshly cleaved mica discs, and allowed to dry overnight at 28 °C before imaging. The surface of *P. entomophila* and *B. thuringiensis* was imaged using NanoScope V AFM (Veeco, USA) in the Analytical Laboratory, Faculty of Chemistry, Maria Curie-Skłodowska University, Lublin, Poland. The measurements were carried out as described in Kordaczuk et al. (70).

### 2.12 Statistical methods

Statistical analysis was performed using Sigma Plot 12.5 (Systat Software Inc., USA). Normality and homoscedasticity of the data were checked with the use of Shapiro-Wilk and Levene’s tests, respectively. When the assumptions of normality and homogeneity of variances were met, parametric tests were performed. If the assumptions were not met, analogous non-parametric tests were performed. For all analysed data, significant differences were established at p < 0.05 in all the tests. In the case of comparison of two groups, the t-test or Mann–Whitney U test were used. Paired t-tests were conducted in the case of comparison of samples of time-dependent data (lysozyme and phenol oxidase activity). Significant differences between two groups of samples were established at *p < 0.05, **p < 0.01, and ***p < 0.001. For comparison of more than two groups, one-way ANOVA or Kruskal-Wallis tests were used, followed by Tukey or Student-Newman-Keuls *post-hoc* tests. To determine differences in the Kaplan-Maier survival curves, Log-rank tests were performed and the P values were indicated in the corresponding figures. All experiments were performed at least three times.

## 3 Results

### 3.1 Priming of *G. mellonella* with *P. entomophila* is specific and results in slowing down the course of future infection

We have shown earlier that injection of 10 P*. entomophila* cells into the hemocel of *G. mellonella* larvae induced the immune response (70). The bacteria were detected in the larval hemocel 8 hours after the injection and in the gut lumen after 24 hours. This was confirmed after the injection of 50 P*. entomophila* cells into the *G. mellonella* hemocel when more bacteria were visible both in the hemocel and in the gut (**Supplementary Figure S1**). This means that *P. entomophila*, even in such a small injection dose, can multiply in the body of an infected insect and reach the gut. Thirty percent of animals injected with 10 P*. entomophila* cells (66) showed external symptoms of infection and further died within 48 hours, but most insects (about 70%) survived.

**Figure 1:**
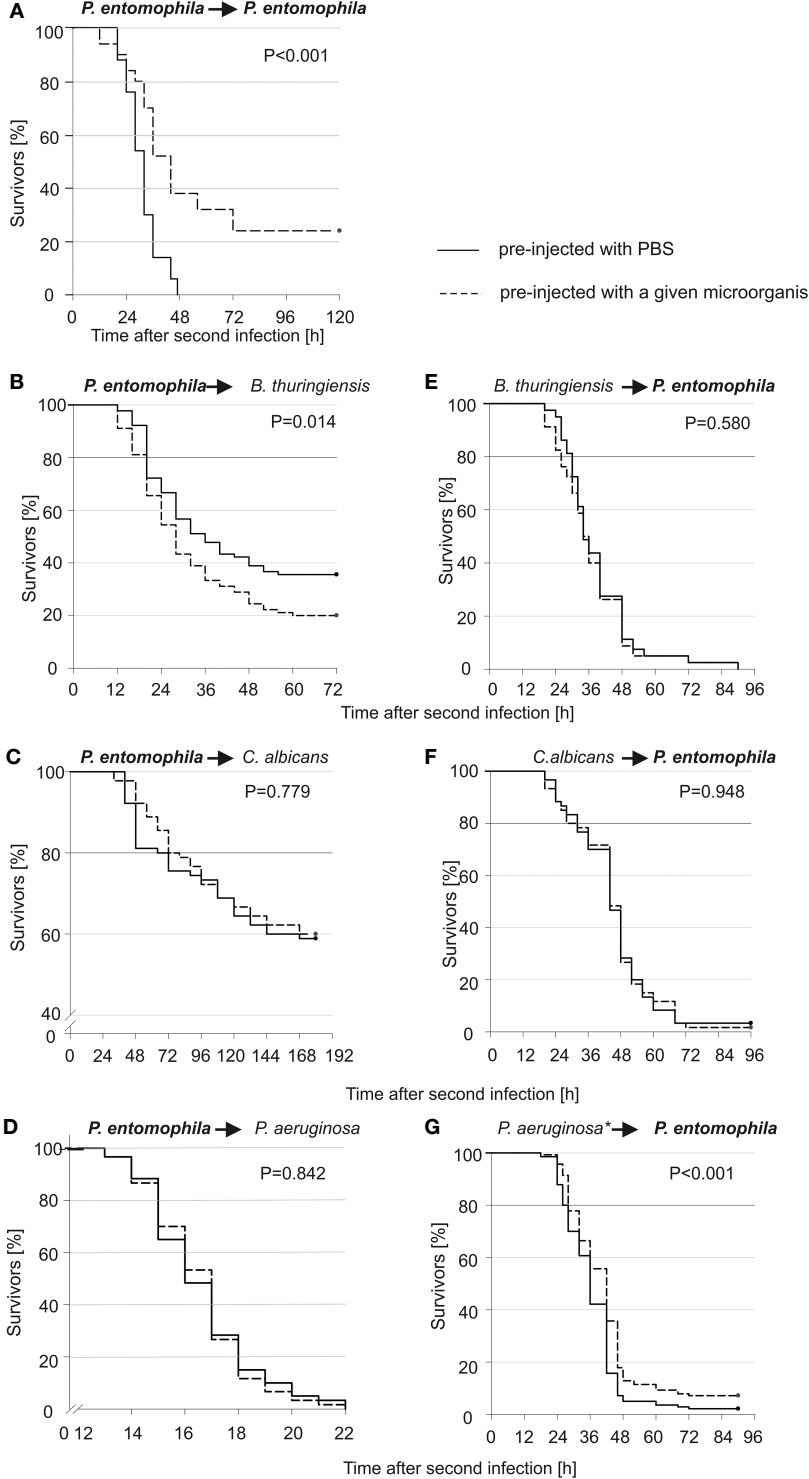
Kaplan-Meier survival analysis of *G. mellonella* larvae after initial infection with 10 *P. entomophila* cells (dotted lines) or with PBS (control, solid lines), followed by infection with different microorganisms (indicated above each graph; left panel), and after initial immune challenge with different microorganisms (indicated above each graph, dotted lines) or with PBS (control, solid lines), followed by infection with 500 *P. entomophila* cells (right panel). The amounts of injected bacteria are provided in the Materials and methods section. The numbers of *G. mellonella* larvae in each group were as follows: 60 **(D, F)**, 80 **(E)**, 90 **(B, C)**, 140 **(G)**, and 50 **(A)**. “*” in case of *P. aeruginosa* stands for heat-killed bacteria.

We checked whether the survivors would be more or less susceptible to re-infection with the same or another microorganism. As can be seen (**Figure 1A**), all larvae that were infected for the first time died within 48 hours, while those pre-infected with 10 P*. entomophila* cells were more resistant to the lethal bacterial dose (500 CFU). After 48 h, about 38% of larvae were still alive and about 25% survived the infection, which was lethal to 100% of the non-primed population. This huge change in the survival curve of the primed and non-primed animals was observed only in the case of re-infection with the same pathogen (compare **Figure 1A** with **Figures 1B–D**). Moreover, after the pre-infection with 10 P*. entomophila* cells, they were more susceptible to the further infection with *B. thuringiensis* (**Figure 1B**). No increase in the resistance to *P. entomophila* was achieved after the pre-infection with *B. thuringiensis* or *C. albicans* (**Figures 1E, F**). Only a minute effect was observed in the case of animals pre-injected with heat-treated *P. aeruginosa* (**Figure 1G**).

**Figure 2:**
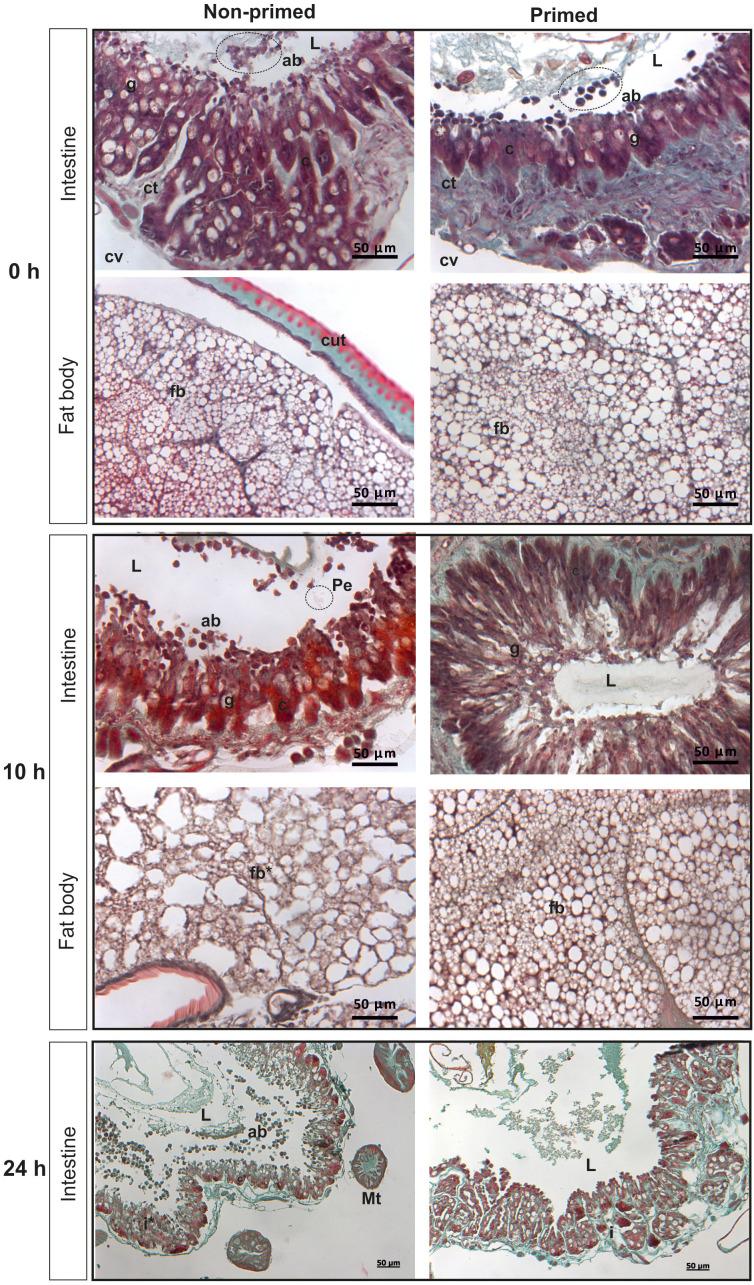
Histological images of cross-section of non-primed and primed *G. mellonella* larvae directly before infection with 500 *P. entomophila* cells (time 0 with respect to priming - 0 h); 10 h and 24 h after injection of 500 P*. entomophila* cells into non-primed and primed *G. mellonella* caterpillars. Images of the intestine and fat body. L, lumen of the intestine; i, intestine; g, Goblet cells; c, columnar cells; cv, body cavity; ab, apoptotic bodies; Pe, *P. entomophila*; ct, contiguous tissue; cut, cuticle; fb, fat body; Mt, Malphigian tubules. Stars denote signs of degradation. Degradation of the fat body and intestine in non-primed larvae at 10 h and 24 h after infection, respectively, is visible.

**Figure 3:**
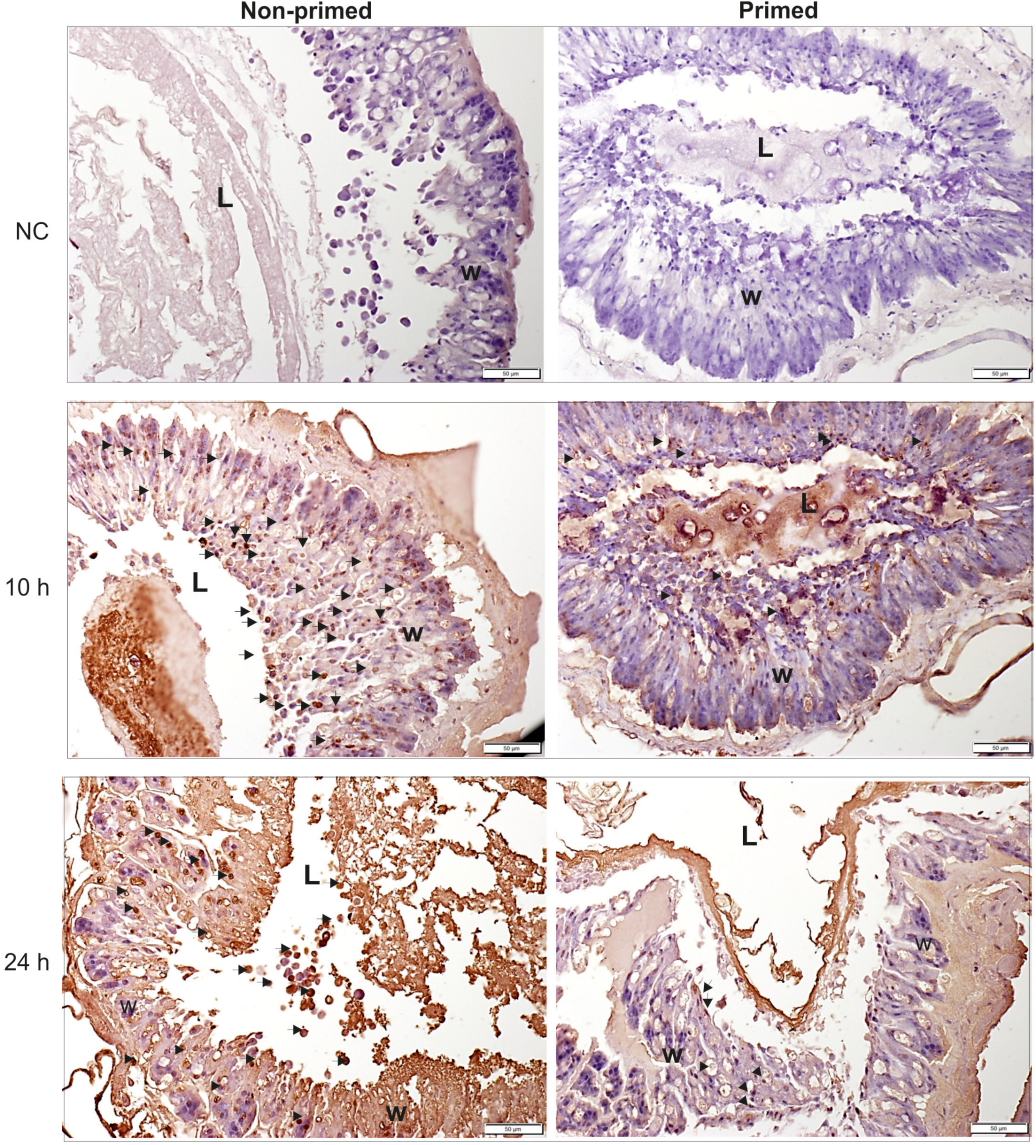
Histological images of cross-section of non-primed and primed *G. mellonella* larvae at 10 h and 24 h after injection of 500 *P. entomophila* cells. The TUNEL reaction was performed to detect apoptotic cells (brown). NC-negative staining control, without the active TdT enzyme, no brown colour is visible. Examples of apoptotic cells are indicated by arrows; L, gut lumen; w, intestine wall.

The analysis of microscopic images showed that the tissues of the non-primed insects and those infected with a low dose did not differ significantly from each other and did not show any signs of past or ongoing infections at the time point preceding the second injection (time 0). At time 0, no bacterial cells were observed in the intestinal lumen or in the body cavity of the primed insects (**Figure 2**). Ten hours after the injection of 500 *P. entomophila* cells, degradation of cells building the fat body was visible in the non-primed insects. Moreover, in these insects, single clusters of bacterial cells in the intestinal lumen were visible. No changes were observed in the case of the primed insects. We noticed that the intestinal structure in the primed insects was even more compact than in the non-primed ones, suggesting a certain renewal (**Figure 2**). Twenty-four hours after the infection, the gut of the non-primed larvae was degraded and macerated, which resulted in weaker coloration. Additionally, atrophy of the gut and a large number of apoptotic cells were observed (**Figure 2**). Apoptotic cells were identified by the TUNEL reaction. As can be seen (**Figure 3**), most of the gut cells of non-primed larvae are apoptotic which is not the case for primed animals. They are located not only on the side of the intestinal lumen (which is physiological) but across the entire width of the intestinal wall.

### 3.2 Most immune parameters are enhanced in primed infected *G. mellonella* larvae

**Figure 4:**
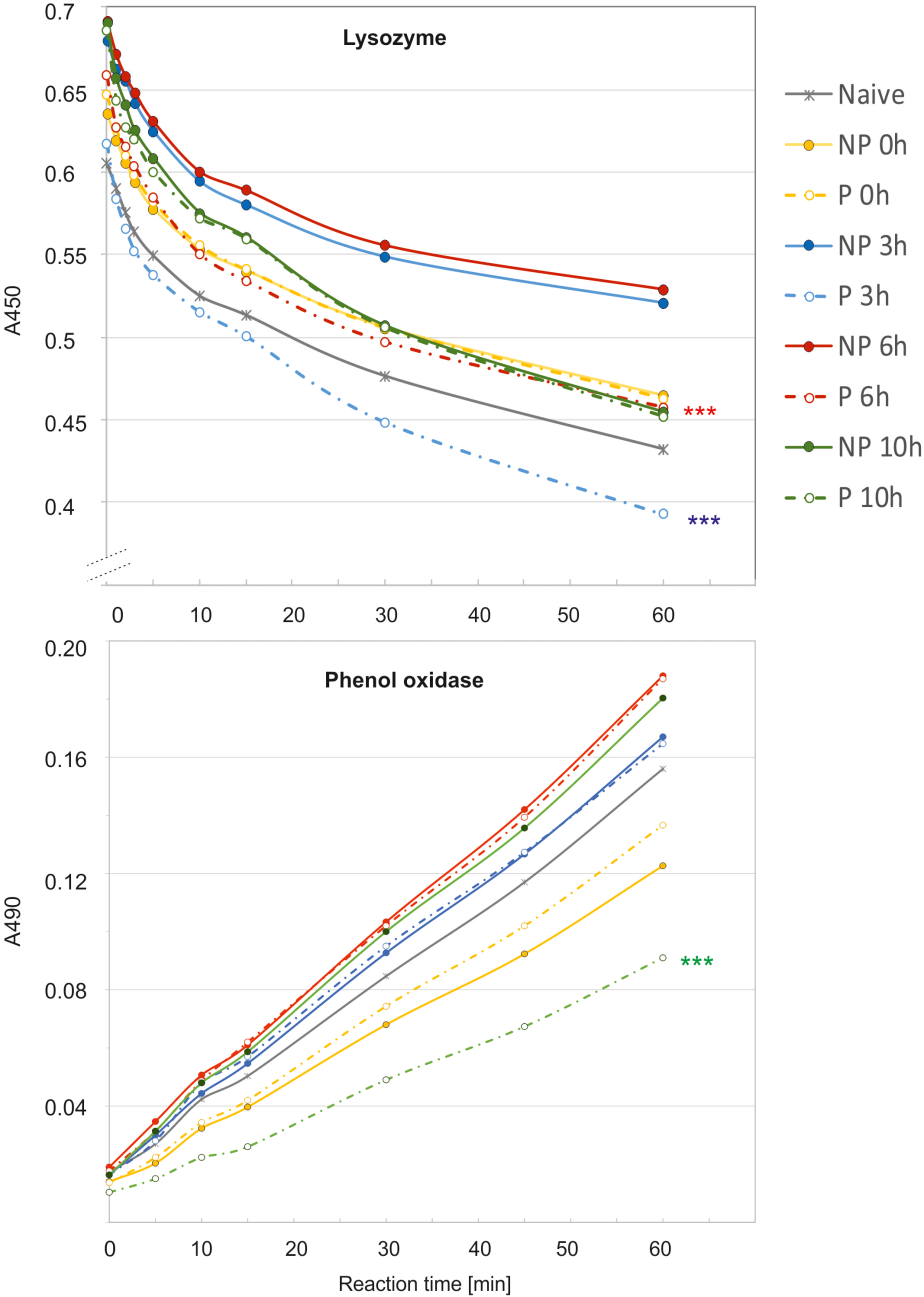
Kinetics of lysozyme (top) and phenol oxidase (bottom) activity in the hemolymph of non-primed (NP, solid lines) and primed (P, dotted lines) *G. mellonella* larvae infected with 500 *P. entomophila* cells. Hemolymph was collected at the indicated time points after infection, as indicated in the legend; 0 h means that hemolymph was collected directly before the infection, i.e. 72 h after the injection of 10 P*. entomophila* cells (P) or PBS (NP); N- naive larvae (solid, grey line). For clarity, results from NP and P pairs from the same time points after infection are lined with the same colour. The decrease in the absorbance (top) shows digestion of peptidoglycan by lysozyme resulting in clearance of the solution, the process which starts very quickly, even before the first measurement (time 0). The increase in the absorbance is (bottom) results of melanin synthesis catalysed by PO present in the hemolymph. Stars, next to the lines for primed group show statistical significance with their corresponding groups of primed larvae (*** P<0.001; n=3; paired t-test).

**Figure 5:**
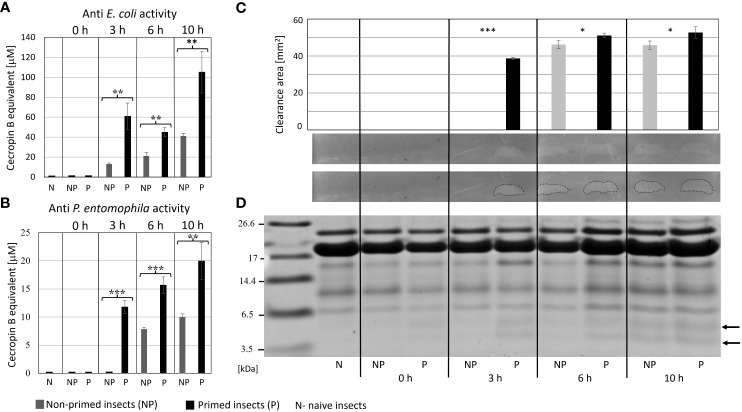
Antimicrobial activity of the hemolymph of non-primed and primed larvae infected with 500 *P. entomophila* cells. Activity of total hemolymph against *E. coli* **(A)** and *P. entomophila* **(B)** and activity of hemolymph proteins separated in polyacrylamide gel **(C)** an original picture and the same gel with marked growth inhibition zones are provided. The graph shows qualitative analysis of growth inhibition zones. Error bars represent SD. Statistical significance between the results from the non-primed (NP) and primed (P) animals are indicated (* P<0.05; ** P<0.01; *** P<0.001, Student t-test, n=3). **(D)** separation of low-molecular weight hemolymph proteins by Tris-tricine SDS-PAGE electrophoresis is shown. Immune-inducible peptides are indicated by arrows.

The analysis of immune parameters, e.g. the antimicrobial activity of hemolymph and gene expression 72 h after the initial infection (time 0 with respect to priming, directly before second infection) revealed that the primed larvae had already turned-off the defence reaction (**Figures 4**, **5**). After the infection of all groups with 500 *P. entomophila* cells, the defence reaction was quicker and stronger in the primed animals in comparison to the non-primed ones. The activity of lysozyme was higher in primed larvae 3 h and 6 h after infection, while activity of phenol oxidase was lower 10 h after infection (**Figure 4**). Increased activity was found against Gram-negative bacteria, which was correlated with the activity of its low-molecular weight components, accompanied by the earlier appearance of peptides below 6 kDa in the hemolymph of the infected animals (**Figure 5**).

### 3.3 Analysis of low-molecular weight peptide components in the hemolymph of non-primed and primed infected larvae

**Figure 6:**
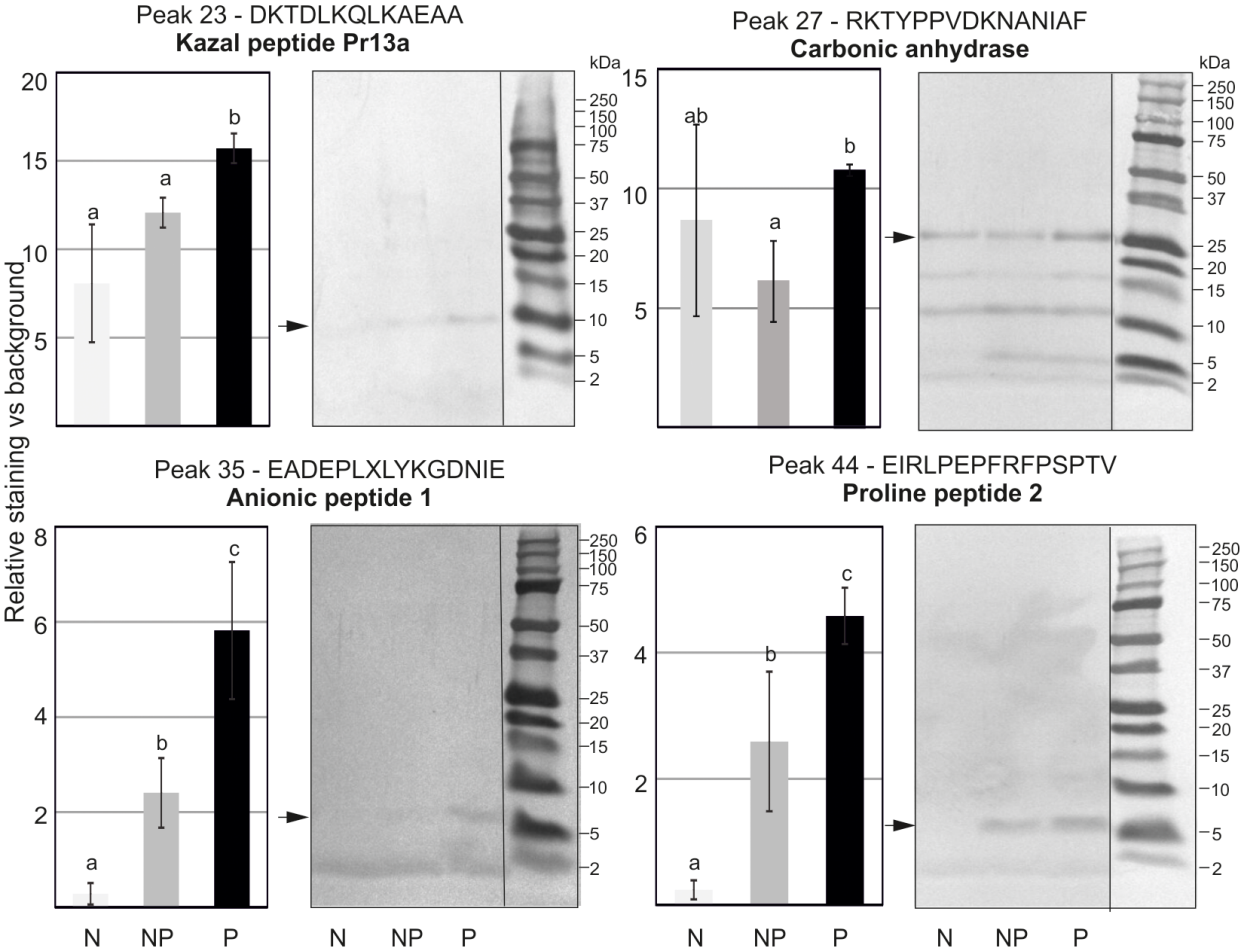
Tris-Tricine electrophoretic analysis of the peptide content in chosen peaks after initial separation by RP-HPLC. The pictures show stained PVDF membranes. Peptides present in different quantities indicated by an asterisk were sequenced and identified. Their names and identified sequences are provided together with the number of peak. Representative pictures are shown, but quantitative densitometric analysis was performed in 3 independent experiments (± SD); N-naive larvae, NP- non-primed larvae infected with 500 P*. entomophila* cells, P-primed larvae infected with 500 *P. entomophila* cells. The differences in staining intensity were estimated with the use of one-way ANOVA. Different letters show statistically significant differences (P<0.05, n=3).

To evaluate the profile of proteins and peptides responsible for the higher antibacterial activity in the primed larvae, methanol extracts containing low-molecular weight polypeptides prepared as described in section 2.7 were separated by RP-HPLC chromatography. The obtained chromatograms consisting of 62 peaks were superimposed on each other, and the area under each peak was measured and analysed to find quantitative differences in the amount of particular peptides or groups of peptides (**Supplementary Figure S2**). We found differences in the level of proteins present in particular fractions. Those showing reproducible differences in the amount of proteins between the primed and non-primed group were further analysed by Tris-Tricine electrophoresis, and particular proteins were identified by N-terminal amino acid sequencing (**Figure 6**). Among proteins whose amount was higher in the primed infected larvae in comparison to the non-primed ones, there were carbonic anhydrase, anionic peptide-1, proline-rich peptide-2, and, so far uncharacterised putative protein XP_026749039.1 named, according to the NCBI database, Kazal peptide Pr13a, whose existence was predicted based on the *G. mellonella* genome sequence.

### 3.4 Priming affects gene expression in re-infected insects

**Figure 7:**
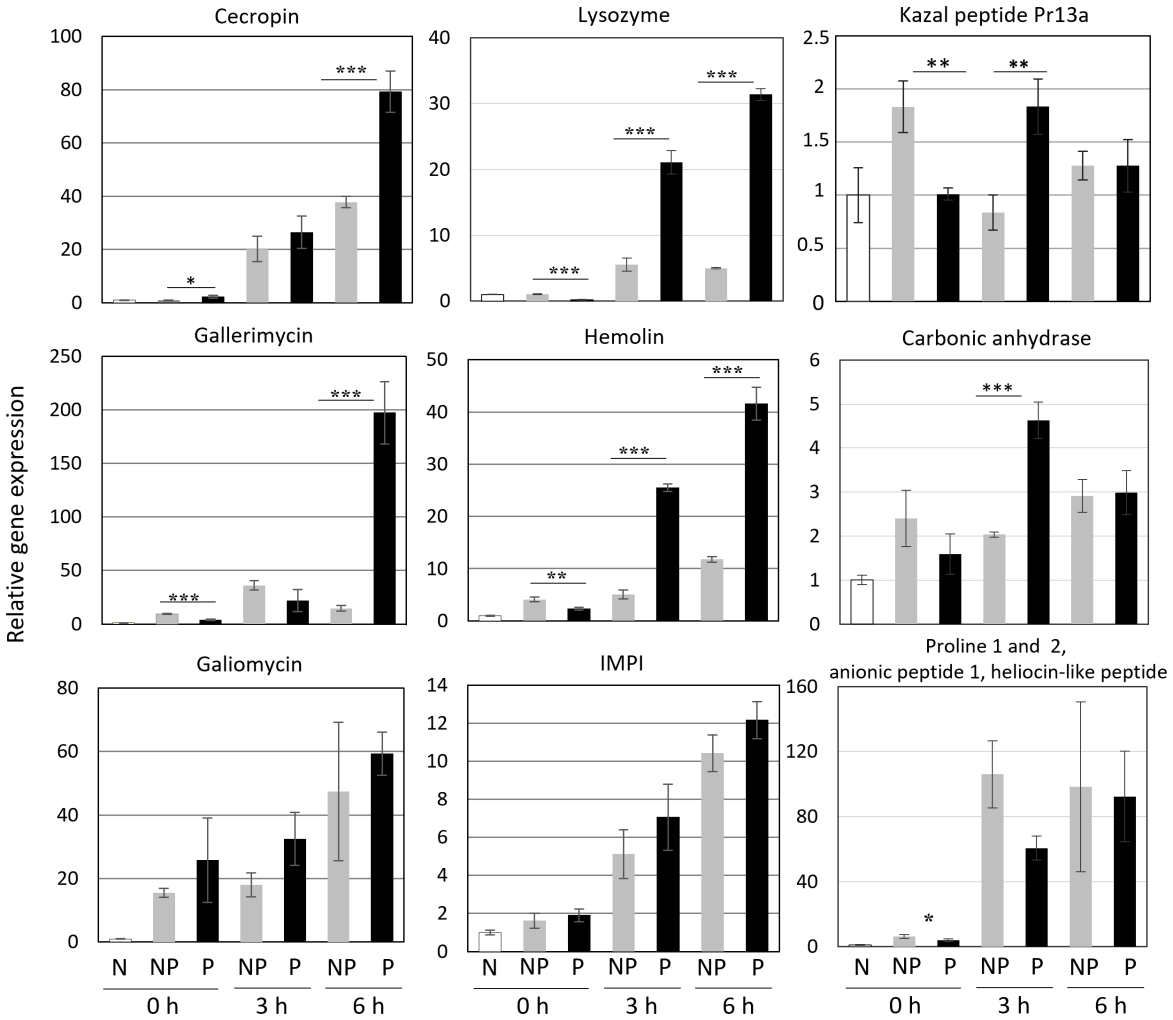
Relative level of expression of genes encoding the indicated peptides and proteins in the fat body of naive (N), non-primed (NP), and primed (P) larvae directly before the infection with 500 CFU of *P. entomophila* (0 h) and at the indicated time points (3 h and 6 h) after the infection. The results are shown in relation to the level of expression of the respective gene in naive larvae (±SD). Asterisks indicate statistical significance (*** P<0.001; ** P<0.01, * P<0.05, Student test, n=3).

We checked the expression of genes encoding proteins present in increased amounts in the hemolymph of re-infected larvae. Additionally, some genes encoding other immune-related proteins were included, i.e. cecropin, gallerimycin, and galiomycin - antimicrobial peptides, hemolin - a pattern recognition receptor (PRR) belonging to the Ig superfamily, and IMPI - an insect metalloproteinase inhibitor. We have found that the expression of some of these genes, i.e. those encoding cecropin, gallerimycin, and hemolin, was enhanced in the re-infected insects. On the other hand, the expression of galiomycin and IMPI was not affected by the priming (**Figure 7**). The expression of genes encoding carbonic anhydrase and the so-far putative Kazal peptide 13a was positively influenced by the priming. Anionic peptide-1 and proline-rich peptides are encoded by one gene (9). Surprisingly, its expression was lower in the infected primed larvae than in the infected non-primed insects (**Figure 7**).

### 3.5 Kazal peptide Pr13a has antimicrobial properties

**Figure 8:**
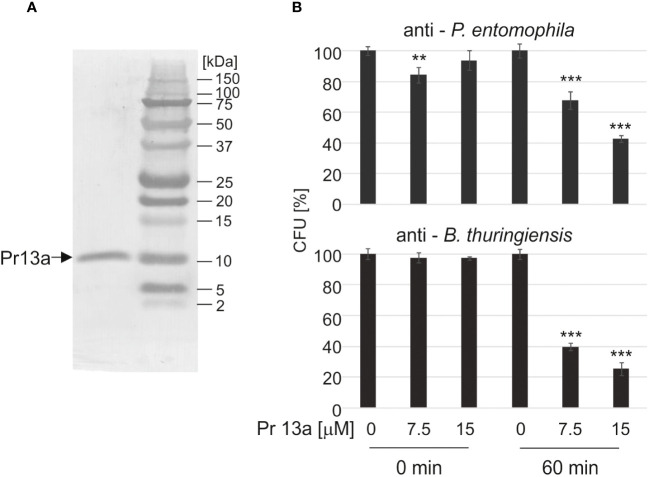
Antimicrobial activity of Kazal peptide Pr13a. The peptide was purified to homogeneity **(A)** and tested at the indicated concentrations for activity against *P. entomophila* and *B. thuringiensis* **(B)** as described in Materials and methods. The control suspension (Time 0) and the suspension after 60-min incubation (Time 60) were plated and the numbers of grown CFU were counted the following day. The results are shown in relation to the number of CFU grown after incubation with water instead of the peptide at the respective time points. Significant differences are indicated (*** P<0.001, ** P< 0.01, Student test, n=6).

Our bioinformatic analysis (see **Supplementary Figure S3**) revealed that Pr13a has homology to vasotab peptide from lesser wax moth *Achroia grisella* and to some Kazal-type serine protease inhibitors (KSPI) although according to NCBI database it does not possess Kazal conserved domains itself. Indeed, we did not detect its inhibiting activity towards serine proteases: trypsin, elastase, and thermolysin metalloproteinase (**Supplementary Figure S4**). To check whether the enhanced expression of the Pr13a gene and the higher amount of the respective protein in the hemolymph of the infected primed larvae may have physiological significance, we checked Pr13a activity against *P. entomophila*. The protein purified to homogeneity from *G. mellonella* hemolymph (**Figure 8A**) had the molecular mass identical to the theoretical one calculated from the gene sequence (10 313 Da, see **Supplementary Figure S3**) and was found to have anti- *P. entomophila* activity (**Figure 8B**). Additionally, we checked the Pr13a activity against another entomopathogenic, Gram-positive bacteria - *Bacillus thuringiensis*. It appeared that Pr13a acted also against this microorganism (**Figure 8B**). It reduced the number of *B. thuringiensis* CFU to about 40% *versus* the control at the concentration of 7.5 µM, while a concentration as high as 15 µM was required for the same reduction of *P. entomophila*. These concentrations were then used during the atomic force microscopy study. We followed changes in the bacterial surface after the incubation of *P. entomophila* and *B. thuringiensis* with Pr13a. We analysed peak force error images showing the topography of bacterial cells, 3D profiles of the bacterial surface, and height images used to prepare surface profiles. Additionally, the roughness and adhesion strength were analysed.

**Figure 9:**
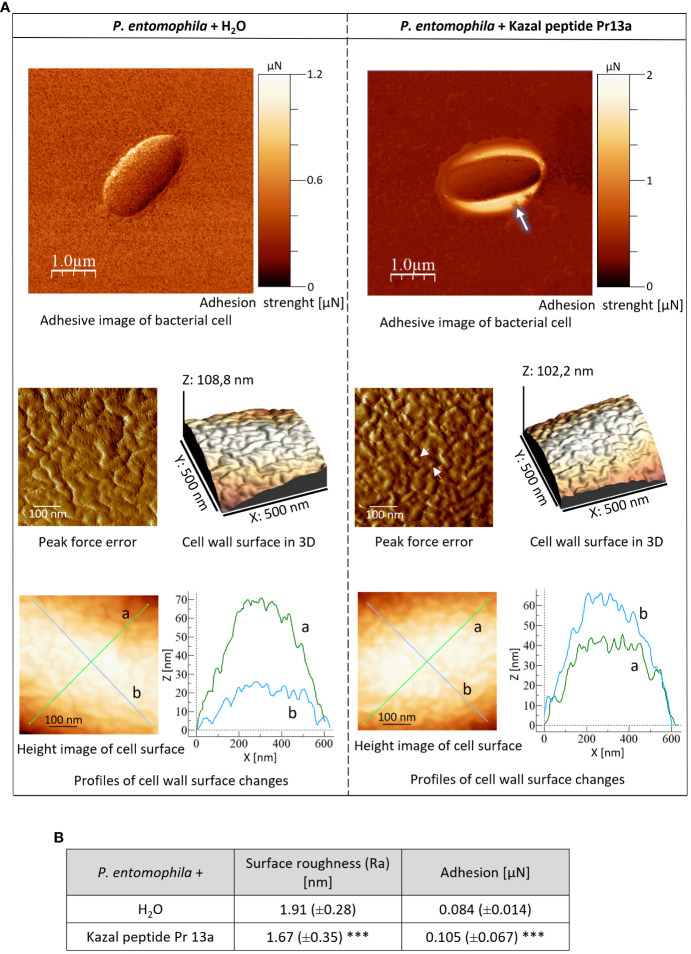
Effect of Kazal peptide Pr13a (15 µM) on *P. entomophila* cell surface topography. Bacteria in the logarithmic growth phase were incubated without (water) or with the Pr13a protein for 1 h Then, the cells were imaged by AFM. **(A)** Adhesive image of whole bacterial cells as well as peak-force-error, three-dimensional, and height images (area 500 nm × 500 nm). Additionally, cell surface change profiles measured along lines a and b marked in the height image are presented on the graphs. **(B)** The table shows biophysical parameters of the cell surface, such as roughness and adhesion force values. Mean values ±SD are shown, n=90. Significant differences are indicated (*** P<0.001, Mann–Whitney U test).

**Figure 10:**
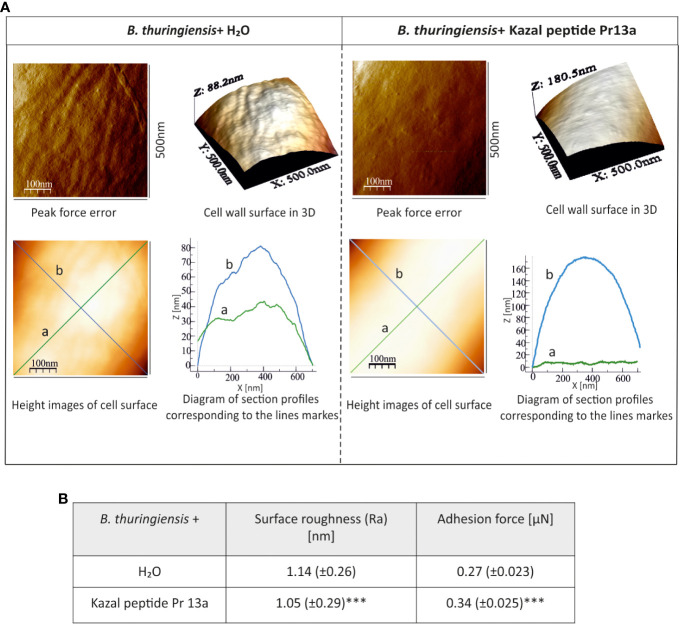
Effect of Kazal peptide Pr13a (7.5 µM) on *B. thuringiensis* cell surface topography. Bacteria in the logarithmic growth phase were incubated without (water) or with the Pr13a protein for 1 h Then, the cells were imaged by AFM. **(A)** Peak-force-error, three-dimensional, and height images (area 500 nm × 500 nm). Additionally, cell surface change profiles measured along lines a and b marked in the height image are presented. **(B)** The table shows biophysical parameters of the cell surface, such as roughness and adhesion force values. Mean values ± SD are shown, n=90. Significant differences are indicated (*** P<0.001, Student t-test and Mann–Whitney U test).

One-hour incubation with the peptide caused loss of bacterial cell firmness, probably as a result of leakage of the cellular contents visible as a bright “halo” around the cell in the adhesive image (**Figure 9A**). The topography analysis carried out on representative fragments of the bacterial cell wall showed numerous hillocks and granules in the peptide-treated cells. The high altitude images revealed the presence of a larger number of higher hillocks (altitude image, Z - coefficient), compared to the control cells, with a very similar highest point on the bacterial surface (108 nm and 102.2 nm in the control and after the incubation with the peptide, respectively). The analysis of biophysical *P. entomophila* parameters revealed significant differences in average cell roughness and adhesion strength between the compared groups (**Figure 9B**). Significant changes were also recorded for the Gram-positive bacteria *B. thuringiensis.* The incubation with 7.5 µM Pr13a resulted in smoothing of the cell surface with the increasing distance between the highest and lowest point (see **Figure 10A**, peak force error and 3-D structure, axis Z). This was confirmed by the high image picture showing that the bigger area is lighter (i.e. higher) but there are fewer hillocks. Indeed, the surface roughness decreased (**Figure 10B**), while the adhesion of the probe to the cell surface increased.

## 4 Discussion

In the presented work, a very strong priming effect on the *G. mellonella* immune system was demonstrated after infection with *P. entomophila*. Priming dose as low as 10 cells per larva was shown before to cause death of 30% animals (66). Re-infection of primed larvae caused not only delay in larval mortality but resulted in a 25% survival rate after the infection with a fifty times higher dose that was 100% lethal to the non-primed insects. This fact eliminates the possibility that the prolonged survival is only a matter of selection. An increase in survival was observed only in the case of repeated infection with the same pathogen. Moreover, the primed insects were even less resistant when infected with *B. thuringiensis*. On the other hand, increased survival against *P. entomophila* was not achieved when the insects were initially infected with *B. thuringiensis* or *C. albicans*, and only a minute effect was observed after the initial immunisation with heat-killed *P. aeruginosa*. This clearly indicates a phenomenon of specific immune priming. Similar specificity was observed in previous studies on *G. mellonella* larvae primed with *B. thuringiensis* or *C. albicans* (41, 43). Larvae initially infected with a low dose of *P. entomophila* and then re-infected with a 50-fold higher dose showed slower infection progress caused by slower colonisation of their body by intruding bacteria. Interestingly, even when the *G. mellonella* were intrahemocelically injected with the bacteria, the microorganisms were quite soon found in the gut lumen. How this bacterium reached the gut lumen after the injection to the hemocel requires further investigations. It is likely that this pathogen prefers the acidic environment of the lumen, where bacterial virulence factors could be activated, especially considering the high resistance of the *P. entomophila* bacteria to acidic pH and reactive oxygen and nitrogen species (52, 71). We observed high degradation of the gut with apoptosis of many cells in the non-primed infected larvae in comparison to the primed ones. Certainly, priming improves the gut structure so it is better protected in re-infected insects.

As stated above, in the case of insects and probably other invertebrates, the phenomenon of immune priming is not common for every host-pathogen system. In the scientific literature, there are suggestions that it might be a lottery whether insects will or will not be more resistant to re-infection. Certainly, this statement resulted from the lack of complete knowledge why infection with a non-lethal dose of a given pathogen will result in an increase in resistance to subsequent infection with the same or another pathogen (36). One possibility that has been emphasised in recent years is that insects develop immunological memory when there is a high probability of re-infection. It occurs in sessile rather than migratory insects and in longer- rather than shorter-living species (72–74). *G. mellonella* is a sessile insect, and its larval stadium takes about 4 weeks. Its larvae are opportunistic cannibals, i.e. they can bite each other in the case of food shortage. Such an injury leads to the formation of an *infection gate* through which various microorganisms can enter the insect’s body cavity (60). Earwax combs are usually stored, e.g. for later melting into wax, in warehouses, where microorganisms have free access and the probability of infection is high. Perhaps this plasticity of its immune system with respect to repeated infection allows this pest to accommodate to its conditions of life (36).

The ability to increase resistance to a specific pathogen but not against others point to the high level of specificity of the *G. mellonella* immune system. We decided to compare the antimicrobial properties of infected primed and non-primed insects and compare their bactericidal peptide profiles. Firstly, we noticed that initially infected and re-infected insects showed a diphasic immune response. It was shown before, that initial injection of 10 P*. entomophila* cells induced the immune response (66). In this paper, we present that 72 h after the initial infection (priming) the immune response was already turned off: the antibacterial activity of hemolymph disappeared and the expression of immune-related genes went back to (at least) the initial level. Moreover, in the initially infected insects, the expression of gallerimycin and hemolin at time 0 (72 hours after initial infection, directly before re-infection) was even lower that in the non-primed group, suggesting inhibition of even the basic expression after the infection was cleared (66 and this paper). This also means that defence mechanisms were re-induced at the time of re-infection. Indeed, when we analysed the expression of genes encoding “markers” of infection, we found the induction of genes encoding cecropin, gallerimycin, galiomycin, hemolin, and IMPI. We noticed enhanced expression of cecropin (antibacterial AMP), gallerimycin (co-operates with cecropin against Gram-negative bacteria), lysozyme (antibacterial, antifungal), and hemolin in the primed larvae in comparison to the non-primed insects. Infection-induced antibacterial activity appeared earlier and was higher in the hemolymph of the primed larvae in comparison to the non-primed ones. It was correlated with the activity of peptides with molecular weight below 10 kDa. The lower activity of PO in the hemolymph of the primed individuals at 10 h post-secondary exposure is also worth mentioning. According to the literature, the PO activity might be hindered by the accumulation of other humoral effectors, especially lysozyme and AMPs (75). The relationship between the higher antibacterial and lysozyme activities in the hemolymph of the primed animals observed 10 h post infection is likely to be the cause of the inhibited PO activity in this group of larvae.

Furthermore, to elucidate the observed antibacterial activity of the hemolymph, methanol extracts containing low-molecular weight protein fractions were subjected to separation using RP-HPLC, and further electrophoretic analysis allowed identification of peptides whose level depended on the priming status of the insects. We found abundant amounts of carbonic anhydrase, anionic peptide- 1, proline-rich peptide-2, and, so far uncharacterised putative protein XP_026749039.1 named Kazal peptide Pr13a in the hemolymph of the primed infected larvae.

Carbonic anhydrases are metabolic enzymes whose main role is to catalyse the reversible hydratation of CO_2_ (76, 77). They are responsible for maintaining the correct pH in insect tissues. Analyses conducted on *B. mori* indicate their key role in the production of cocoon silk (78). A recent report on the metabolic approach to the phenomenon of innate memory in *G. mellonella* documented that immune priming of larvae with *B. thuringiensis* leads to changes in 130 different metabolites. These changes are a result of the regulation of metabolic pathways involved in the biosynthesis of amino acids, aminoacyl-tRNA, and carbon metabolism (79). The observed differences in the expression of genes correlated with protein contents between primed and non-primed infected *G. mellonella* may be a result of imprinting the “metabolic mark” that determines the presence of some kind of innate immune memory or helps the host to cope with the consequences of former infection and its subsequent development (79, 80). Anionic peptide-1 and proline rich peptide-2 are known antimicrobial peptides found in *G. mellonella* hemolymph. They were reported to have anti-bacterial activity (8). Their higher amount in the hemolymph of primed larvae is correlated with the higher antibacterial activity of cell-free hemolymph and is probably a result of the regulatory mechanism standing behind innate memory. Interestingly, anionic peptide-2 and proline rich peptide-1, together with proline rich peptide-2 and heliocin-like peptide, are encoded by one gene (9). Its expression was not higher in the primed larvae than in the non-primed ones. A higher amount of anionic peptide-1 and proline rich peptide-2 in the hemolymph of primed infected larvae could be a result of post-transcription events. Possibly, priming results in specialisation of ribosomes, which preferably use the transcripts for proline-rich peptides and anionic peptide-2, resulting in a higher amount of the respective proteins (81, 82). This mechanism would increase the ergonomy of the re-induced immune response, because transcription is an energetically costly process. Saving energy would probably be possible as well if mRNA or the proteins themselves were more stable in the hemolymph. In the case of proteins, the stability could be achieved thanks to chaperones, whose amount increases in stress conditions that are certainly created during infection. Indeed, both heat shock and infection result in a higher amount of Hsp90 (83). This possibility is enhanced by the fact that, in the case of *G. mellonella* priming with *B. thuringiensis*, the expression of some genes encoding antimicrobial peptides was not enhanced in the primed insects despite the higher antimicrobial activity of the hemolymph (41).

On the other hand, the expression of some genes encoding low-molecular compounds, e.g. lysozyme, was higher in the primed infected larvae, but the respective protein was not present in increased amounts in the hemolymph. We observed increased gene expression of the lysozyme-encoding gene, but did not detect an increased amount of the protein in the hemolymph, as lysozyme may be bound to bacteria. Indeed, cecropin and lysozyme have direct anti-*P. entomophila* activity (66). On the other hand, we detected higher lysozyme activity in the hemolymph of the primed infected *G. mellonella* than in the non-primed larvae. This means that the activity of lysozyme could be regulated by, e.g., some other peptides. Cytryńska et al. (84) found that lysozyme acts synergistically with antimicrobial peptides. Its increased activity may also be a result of lower activity of phenyl oxidase (as shown in this paper), as there are reports that the activity of one protein has a negative effect on the activity of the other (75). Another reason could be that lysozyme is a target for the action of *P. entomophila* virulence factors. When *G. mellonella* were fed with 10^5^ CFU of *P. entomophila*, the amount of lysozyme was more than 50% reduced in comparison to its amount after infection with a lower 10^3^ CFU dose. This was correlated with disappearance of antibacterial properties of the hemolymph (66). Probably, our observations are a result of all the possibilities presented above.

Finally, we found Kazal peptide Pr13a to be one of immune-induced proteins whose amount was higher in the primed larvae than in the non-primed ones. The expression of the Pr13 gene after the single injection of 10 and 50 *P. entomophila* cells was about twice as high as after the injection of PBS (data not shown). Then, 3 h after the infection, it was twice as high in the primed larvae as in the non-primed insects.

The Pr13a peptide is a putative protein with sequence similarity to Kazal-type inhibitors of proteases. The sequence analysis indicated its similarity to the putative vasotab peptide from the lesser wax moth *Achroia grisella* and to the protease inhibitor-like protein from *Antheraea mylitta*. The further bioinformatic analysis did not reveal any characteristic conserved domains that can classify this peptide into any protein family. This also concerns Kazal domains, which are absent in Pr13a (NCBI database). This is consistent with the fact that this peptide did not inhibit proteolytic enzymes, e.g. thermolysin, trypsin, or elastase (**Supplementary Figure S4**). There is no other information in the literature regarding peptide Pr13a, and its existence has so far been only alleged.

We checked whether the quantitative differences between non-primed and primed larvae after infection with a high *P. entomophila* dose would have physiological meaning. It appeared that Pr13a has direct anti-*P. entomophila* activity. At the concentration of 15 µM, it reduced the number of *P. entomophila* cells to about 40%. Moreover, the AFM analysis showed leakage of intracellular fluids outside the cells and changes in the 3D structure of the bacterial surface: a decrease in roughness and a slight increase in adhesion properties. The observed changes may result from the interaction of the tested protein with elements that constitute the bacterial cell envelope, leading to changes in the permeability and structure of cell membranes (75, 85). We also detected anti- *B. thuringiensis* activity of Kazal peptide Pr13a. At the concentration of 7.5 µM, it reduced the number of CFU to about 40%. This was correlated with changes detected on the bacterial surface, such as a decrease in roughness and an increase in adhesion of the probe to the bacterial surface. Changes in the features of the bacterial surface may be induced by, e.g., cecropin D from *G. mellonella*, which binds LPS and other lipid components building the *E. coli* surface. Cecropin D is incorporated and leads to structural and physicochemical changes in bacterial envelopes (86).

The presented results clearly indicate the potential contribution of immune peptides to the phenomenon of innate memory in the *G. mellonella - P. entomophila* system. It is worth emphasising that the most powerful increase in resistance was observed only in the homologous system; this may indicate that this extremely sophisticated adaptive mechanism is able to act faster and more efficiently, triggering re-induction of the immune response prior to silencing the gene expression process and only after a specific stimulus.

## Data availability statement

The original contributions presented in the study are included in the article/**Supplementary Material**. Further inquiries can be directed to the corresponding author.

## Ethics statement

The manuscript presents research on animals that do not require ethical approval for their study.

## Author contributions

MS: Investigation, Writing – review & editing. JK: Investigation, Writing – review & editing. PM: Investigation, Methodology, Writing – review & editing. JS-B: Investigation, Writing – review & editing. MH-S: Investigation, Writing – review & editing. IW: Conceptualization, Funding acquisition, Project administration, Supervision, Writing – original draft, Writing – review & editing.

## References

[1] Wojda I. Immunity of the greater wax moth Galleria mellonella. Insect Sci. (2017) 24(3):342–57. doi: 10.1111/1744-7917.12325 26847724

[2] Hetru C Hoffmann JA. NF-kappaB in the immune response of Drosophila. Cold Spring Harb Perspect Biol. (2009) 1:a000232. doi: 10.1101/cshperspect.a000232 20457557 PMC2882123

[3] Hillyer JF. Insect immunology and hematopoesis. Dev Comp Immunol. (2016) 58:102–18. doi: 10.1016/j.dci.2015.12.006 PMC4775421 26695127

[4] Ono M Yoshiga T. Cellular immunity in the insect Galleria mellonella against insect non-parasitic nematodes. Parasitology. (2019) 146(6):708–15. doi: 10.1017/S003118201800210X 30567609

[5] Serrano I Verdial C Tavares L Oliveira M. The virtuous *Galleria mellonella* model for scientific experimentation. Antibiotics. (2023) 12(3):505. doi: 10.3390/antibiotics12030505 36978373 PMC10044286

[6] Chen RY Keddie BA. *Galleria mellonella* (Lepidoptera: Pyralidae) Haemocytes release extracellular traps that confer protection against bacterial infection in the hemocoel. J Insect Sci. (2021) 21:17. doi: 10.1093/jisesa/ieab092 PMC8643984 34865034

[7] Eleftherianos I Revenis C. Role and importance of phenoloxidase in insect hemostasis. J Innate Immun. (2011) 3(1):28–33. doi: 10.1159/000321931 21051882

[8] Cytryńska M Mak P Zdybicka-Barabas A Suder P Jakubowicz T. Purification and characterization of eight peptides from *Galleria mellonella* immune hemolymph. Peptides. (2007) 28:533–46. doi: 10.1016/j.peptides.2006.11.010 17194500

[9] Brown SE Howard A Kasprzak AB Gordon KH East PD. A peptidomics study reveals the impressive antimicrobial peptide arsenal of the wax moth *Galleria mellonella* . Insect Biochem Mol Biol. (2009) 39(11):792–800. doi: 10.1016/j.ibmb.2009.09.004 19786100

[10] Kim CH Lee JH Kim I Seo SJ Son SM Lee KY. Purification and cDNA cloning of a cecropin-like peptide from the great wax moth, *Galleria mellonella* . Mol Cells. (2004) 17(2):262–6. doi: 10.1016/S1016-8478(23)13036-6 15179040

[11] Schuhmann B Seitz V Vilcinskas A Podsiadlowski L. Cloning and expression of gallerimycin, an antifungal peptide expressed in immune response of greater wax moth larvae, Galleria mellonella. Arch Insect Biochem Physiol. (2003) 53:125–33. doi: 10.1002/arch.10091 12811766

[12] Seitz V Clermont A Wedde M Hummel M Vilcinskas A Schlatterer K. Identification of immunorelevant genes from greater wax moth (*Galleria mellonella*) by a subtractive hybridization approach. Dev Comp Immunol. (2003) 27(3):207–15. doi: 10.1016/s0145-305x(02)00097-6 12590972

[13] Lee YS Yun EK Jang WS Kim I Lee JH Park SY. Purification, cDNA cloning and expression of an insect defensin from the great wax moth, *Galleria mellonella* . Insect Mol Biol. (2004) 13(1):65–72. doi: 10.1111/j.1365-2583.2004.00462.x 14728668

[14] Park SY Kim CH Jeong WH Lee JH Seo SJ Han YS. Effects of two hemolymph proteins on humoral defense reactions in the wax moth, *Galleria mellonella* . Dev Comp Immunol. (2005) 29(1):43–51. doi: 10.1016/j.dci.2004.06.001 15325522

[15] Lemaitre B Kromer-Metzger E Michaut L Nicolas E Meister M Georgel P. A recessive mutation, immune deficiency (imd) defines two distinct control pathways in the *Drosophila* host defence. Proc Natl Acad Sci USA. (1995) 92(21):9465–9. doi: 10.1073/pnas.92.21.9465 PMC40822 7568155

[16] Lemaitre B Nicolas E Michaut L Reichhart J-M Hoffmann JA. The dorsoventral regulatory gene cassette spatzle/toll/cactus controls the potent antifungal response in *Drosophila* adults. Cell. (1996) 86:973–83. doi: 10.1016/S0092-8674(00)80172-5 8808632

[17] Silverman N Paquette N Aggarwal K. Specificity and signalling in the *Drosophila* immune response. Invert Surv J. (2009) 6:163–74. PMC3101770 21625362

[18] Sowa-Jasiłek A Zdybicka-Barabas A Stączek S Wydrych J Mak P Jakubowicz T. Studies on the role of insect hemolymph polypeptides: *Galleria mellonella* anionic peptide 2 and lysozyme. Peptides. (2014) 53:194–201. doi: 10.1016/j.peptides.2014.01.012 24472857

[19] Bolouri Moghaddam MR Tonk M Schreiber C Salzig D Czermak P Vilcinskas A. The potential of the *Galleria mellonella* innate immune system is maximized by the co-presentation of diverse antimicrobial peptides. Biol Chem. (2016) 397(9):939–45. doi: 10.1515/hsz-2016-0157 27105487

[20] Cytryńska M Wojda I Jakubowicz T. ”How insects combat infections”. In: Ballarin L Cammarata M, editors. Lessons in Immunity. Cambridge, MA: Academic Press (2016). p. 117–28.

[21] Metalnikow S. Immunit´e naturelle ou acquise des chenilles de *Galleria mellonella* . C R Acad Sci. (1920) 83:817–20.

[22] Paillot A. L’Infection chez les insects. Immunité et Symbiose. Bulletin de la Société entomologique de France (1933) 38(18):295–6.

[23] Boman HG Nilsson I Rasmuson B. Inducible antibacterial defence in. Drosophila. Nature. (1972) 237:232–5. doi: 10.1038/237232a0 4625204

[24] Brey PT. The contributions of Pasteur school of insect immunology. Molecular mechanisms of immune responses in insects. London: Chapman & Hall (1998) p. 1–39.

[25] Ligoxygakis P. Immunity: Insect immune memory goes viral. Curr Biol. (2017) 27:1218–20. doi: 10.1016/j.cub.2017.10.020 29161560

[26] Little TJ Kraaijeveld AR. Ecological and evolutionary implications of immunological priming in invertebrates. Trends Ecol Evol. (2004) 19:58–60. doi: 10.1016/j.tree.2003.11.011 16701227

[27] Bergin D Murphy L Keenan J Clynes M Kavanagh K. Pre-exposure to yeast protects larvae of *Galleria mellonella* from a subsequent lethal infection by *Candida albicans* and is mediated by the increased expression of antimicrobial peptides. Microbes Infect. (2006) 8:2105–12012. doi: 10.1016/j.micinf.2006.03.005 16782387

[28] Wu G Yi Y Lv Y Li M Wang J Qiu L. The lipopolysaccharide (LPS) of *Photorhabdus luminescens* TT01 can elicit dose- and time-dependent immune priming in *Galleria mellonella* larvae. J Invert Pathol. (2015) 127:63–72. doi: 10.1016/j.jip.2015.03.007 25796336

[29] Wu G Zhao Z Liu C Qiu L. Priming *Galleria mellonella* (Lepidoptera: Pyralidae) larvae with heat-killed bacterial cells induced an enhanced immune protection against *Photorhabdus luminescens* TT01 and the role of innate immunity in the process. J Econ Ento-mol. (2014) 107:559–69. doi: 10.1603/EC13455 24772535

[30] Coustau C Kurtz J Moret YA. Novel mechanism of immune memory unveiled at the invertebrate-parasite interface. Trends Parasitol. (2016) 32:353–5. doi: 10.1016/j.pt.2016.02.005 26953517

[31] Milutinović B Kurtz J. Immune memory in invertebrates. Semin Immunol. (2016) 28:328–42. doi: 10.1016/j.smim.2016.05.004 27402055

[32] Milutinović B Peuß R Ferro K Kurtz J. Immune priming in arthropods: an update focusing on the red flour beetle. Zoology. (2016) 119:254–61. doi: 10.1016/j.zool.2016.03.006 27350318

[33] Pinaud S Portela J Duval D Nowacki FC Olive MA Allienne J-F. A shift from cellular to humoral responses contributes to innate immune memory in the vector snail *Biomphalaria glabrata* . PloS Pathog. (2016) 12(1):e1005361. doi: 10.1371/journal.ppat.1005361 26735307 PMC4703209

[34] Brehélin M Roch P. Specificity, learning and memory in the innate immune response. Invert Surv Journ. (2008) 5:103–9.

[35] Contreras-Garduño J Carmen Rodríguez M Hernandez-Martínez S MartínezBarnetche J Alvarado-Delgado A Izquierdo J. *Plasmodium berghei* induced priming in *Anopheles albimanus* independently of bacterial co-infection. Dev Comp Immunol. (2015) 52:172–81. doi: 10.1016/j.dci.2015.05.004 26004500

[36] Contreras- Garduño J Lanz-Mendoza H Franco B Nava A Pedraza- Reyes M Canales-Lazcano J. Insect immune priming: ecology and experimental evidences. Ecol Entomol. (2016) 41:351–66. doi: 10.1111/een.12300

[37] Kurtz J. Specific memory with in innate immune systems. Trends Immunol. (2005) 24:186–92. doi: 10.1016/j.it.2005.02.001 15797508

[38] Schmid-Hempel P. Evolutionary parasitology. Oxford: Oxford University Press (2011).

[39] Faulhaber LM Karp RD. A diphasic immune response against bacteria in the american cockroach. Immunology. (1992) 75:378–81. PMC1384723 1551700

[40] Sadd BM Schmid-Hempel P. Insect immunity shows specificity in protection upon secondary pathogen exposure. Curr Biol. (2006) 16:1206–10. doi: 10.1016/j.cub.2006.04.047 16782011

[41] Taszłow P Vertyporokh L Wojda I. Humoral immune response of *Galleria mellonella* after repeated infection with *Bacillus thuringiensis* . J Invertebr Pathol. (2017) 149:87–96. doi: 10.1016/j.jip.2017.08.008 28803980

[42] Medina-Gomez H Rivas GA Hernandez-Quintero A Gonzalez - Hernandez A Torres Guzm´ an JC Mendoza HL. The occurrence of immune priming can be species-specific in entomopathogens. Microb Pathog. (2018) 118:361–4. doi: 10.1016/j.micpath.2018.03.063 29614365

[43] Vertyporokh L Kordaczuk J Hułas-Stasiak M Mak P Wojda I. Host-pathogen interaction upon the first and subsequent infection of *Galleria mellonella* with *Candida albicans* . J Insect Physiol. (2019) 117:103903. doi: 10.1016/j.jinsphys.2019.103903 31233768

[44] Roth O Kurtz J. Phagocytosis mediates specificity in the immune defence of an invertebrate, the woodlouse *Porcellio scaber* (Crustacea: Isopoda). Dev Comp Immunol. (2009) 33:1151–5. doi: 10.1016/j.dci.2009.04.005 19416736

[45] Ferro K Peuß R Yang W Rosenstiel P Schulenburg H Kurtz J. Experimental evolution of immunological specificity. PNAS. (2019) 116:20598–604. doi: 10.1073/pnas.1904828116 PMC6789748 31548373

[46] Li W. Dscam in arthropod immune priming: What is known and what remains unknown. Dev Comp Immunol. (2021) 125:104231. doi: 10.1016/j.dci.2021.104231 34390752

[47] Freitak D Schmidtberg H Dickel F Lochnit G Vogel H Vilcinskas A. The maternal transfer of bacteria can mediate trans-generational immune priming in insects. Virulence. (2014) 5(4):547–54. doi: 10.4161/viru.28367 PMC4063815 24603099

[48] Vilcinskas A. The role of epigenetics in host-parasite coevolution: lessons from the model host insects *Galleria mellonella* and Tribolium castaneum. Zoology (Jena). (2016) 119(4):273–80. doi: 10.1016/j.zool.2016.05.004 27341739

[49] Vilcinskas A. Mechanisms of transgenerational immune priming in insects. Dev Comp Immunol. (2021) 124:104205. doi: 10.1016/j.dci.2021.104205 34260954

[50] Vodovar N Vinals M Liehl P Spellman P Boccard F Lemaitre B. *Drosophila* host defense after oral infection by an entomopathogenic *Pseudomonas* species. Proc Natl Acad Sci USA. (2005) 102(32):1414–11419. doi: 10.1073/pnas.0502240102 PMC1183552 16061818

[51] Vodovar N Vallenet D Cruveille S Rouy Z Barbe V Acosta C. Complete genome sequence of the entomopathogenic and metabolically versatile soil bacterium *Pseudomonas entomophila* . Nat Biotechnol. (2006) 24(6):673–9. doi: 10.1038/nbt1212 16699499

[52] Dieppois G Opota O Lalucat J Lemaitre B. *Pseudomonas entomophila*: A versatile bacterium with entomopathogenic properties. New aspects Pseudomonas Biol. (2015) 7:25–50. doi: 10.1007/978-94-017-9555-5_2

[53] Mulet M Gomila M Lemaitre B Lalucat J Garcia–Valdes E. Taxonomic characterisation of *Pseudomonas* strain L48 and formal proposal of *Pseudomonas entomophila* sp. Nov Syst Appl Microbiol. (2012) 35:145–9. doi: 10.1016/j.syapm.2011.12.003 22326814

[54] Liehl P Blight M Vodovar N Boccard F Lemaitre B. Prevalence of local immune response against oral infection in a Drosophila/Pseudomonas infection model. PloS Pathog. (2006) 2:e56. doi: 10.1371/journal.ppat.0020056 16789834 PMC1475658

[55] Opota O Vallet–Gely I Vincentelli R Kellenberger C Iacovache I Gonzalez MR. Monalysin, a novel ss– pore–forming toxin from the Drosophila pathogen Pseudomonas entomophila, contributes to host intestinal damage and lethality. PloS Pathog. (2011) 7:e1002259. doi: 10.1371/journal.ppat.1002259 21980286 PMC3182943

[56] Sarris PF Scoulica EV. *Pseudomonas entomophila* and *Pseudomonas mendocina*: potential models for studying the bacterial type VI secretion system. Infect Genet Evol. (2011) 11:1352–60. doi: 10.1016/j.meegid.2011.04.029 21600307

[57] Nonaka S Salim E Kamiya K Hori A Nainu F Asri RM. Molecular and functional analysis of pore–forming toxin monalysin from entomopathogenic bacterium Pseudomonas entomophila. Front Immunol. (2020) 27:511–20. doi: 10.3389/fimmu.2020.00520 PMC7118224 32292407

[58] Vallet–Gely I Novikov A Augusto L Liehl P Bolbach G Pechy-Tarr M. Association of hemolytic activity of *Pseudomonas entomophila*, a versatile soil bacterium, with cyclic lipopeptide productio. Appl Environ Microbiol. (2010) 76:910–21. doi: 10.1128/AEM.02112-09 PMC2812987 20023108

[59] Omoboye OO Oni FE Batool H Yimer HZ De Mot R Höfte M. *Pseudomonas* cyclic lipopeptides suppress the rice blast fungus *Magnaporthe oryzae* by induced resistance and direct antagonism. Front Plant Sci. (2019) 10:901. doi: 10.3389/fpls.2019.00901 31354771 PMC6636606

[60] Wojda I Cytryńska M Zdybicka-Barabas A Kordaczuk J. Insect defense proteins and peptides. Subcell Biochem. (2020) 94:81–121. doi: 10.1007/978-3-030-41769-7_4 32189297

[61] Goel MK Khanna P Kishore J. Understanding survival analysis: Kaplan-Meier estimate. Int J Ayurv Res. (2010) 1:274–8. doi: 10.4103/0974-7788.76794 PMC3059453 21455458

[62] Luna LG. Histopathologic Methods and Color Atlas of Special Stains and Tissue Artifacts. Gaithersburg: Am Histolabs (1992).

[63] Boman HG Nilsson-Faye I Paul K Rasmuson T Jr.. Insect immunity. I. Characteristics of an inducible cell- free antibacterial reaction in hemolymph of Samia cythia pupae. Infect Immun. (1974) 10:136–45. 10.1128/iai.10.1.136-145.1974 PMC414969 4210336

[64] Hultmark D. “Quantification of antimicrobial activity using the inhibition- zone assay”. In: Wiesner A Dunphy GB Marmaras VJ Morishima I Sugumaran M, editors. Techniques in insect immunology. Fair Haven, NJ: SOS Publications (1998). p. 103–7. doi: 10.1093/nar/29.9.e45

[65] Laemmli UK. Cleavage of structural proteins during the assembly of the head of bacteriophage T4. Nature. (1970) 227:680–5. 10.1038/227680a0 5432063

[66] Kordaczuk J Sułek M Mak P Śmiałek-Bartyzel J Hułas-Stasiak M Wojda I. Defence response of *Galleria mellonella* larvae to oral and intrahemocelic infection with *Pseudomonas entomophila* . Dev Comp Immunol. (2023) 147:104749. doi: 10.1016/j.dci.2023.104749 37279831

[67] Livak KJ Schmittgen TD. Analysis of relative gene expression data using real time quantitative PCR and the 2(–delta delta C(T)) method. Methods. (2001) 25:402–8. doi: 10.1006/meth.2001.1262 11846609

[68] Pfaffl MW. A new mathematical model for relative quantification in real-time RTPCR. Nucleic Acids Res. (2001) 29:e45. 11328886 10.1093/nar/29.9.e45 PMC55695

[69] Schagger H von Jagow G. Tricine-sodium dodecyl sulfate-polyacrylamide gel electrophoresis for the separation of proteins in the range from 1 to 100 kDa. Analytical Biochem. (1987) 166:368–79. doi: 10.1016/0003-2697(87)90587-2 2449095

[70] Kordaczuk J Sułek M Mak P Zdybicka-Barabas A Śmiałek J Wojda I. Cationic protein 8 plays multiple roles in *Galleria mellonella* immunity. Sci Rep. (2022) 12(1):11737. doi: 10.1038/s41598-022-15929-6 35817811 PMC9273619

[71] Chakrabarti S Liehl P Buchon N Lemaitre B. Infection-induced host translational blockage inhibits immune responses and epithelial renewal in the *Drosophila* gut. Cell Host Microbe. (2012) 12(1):60–70. doi: 10.1016/j.chom.2012.06.001 22817988

[72] Best A Tidbury H White A Boots M. The evolutionary dynamics of within-generation immune priming in invertebrate hosts. J R Soc Interface. (2013) 10:20120887. doi: 10.1098/rsif.2012.0887 23269850 PMC3565738

[73] Pigeault R Garnier R Rivero A Gandon S. Evolution of transgenerational immunity in invertebrates. Proc Biol Sci. (2016) 283:20161136. doi: 10.1098/rspb.2016.1136 27683366 PMC5046895

[74] Moret Y Coustau C Braquart-Varnier C Gourbal B. “Immune priming and trans-generational protection from parasites”. In: Choe JC, editor. Encyclopedia of animal behavior. Cambridge, MA: Academic Press (2019). p. 764–74. doi: 10.1016/B978-0-12-809633-8.90726-X

[75] Zdybicka-Barabas A Palusińska-Szysz M Gruszecki WI Mak P Cytryńska M. *Galleria mellonella* apolipophorin III – an apolipoprotein with anti-*Legionella pneumophila* activity. Biochim Biophys Acta (BBA) - Biomembranes. (2014) 1838(10):2689–97. doi: 10.1016/J.BBAMEM.2014.07.003 25016052

[76] Burt E Darlington MV Graf G Meyer HJ. Isolation, purification and characterization of an insect carbonic anhydrase. Insect Biochem Mol Biol. (1992) 22(3):285–91. doi: 10.1016/0965-1748(92)90066-N

[77] Haritos VS Dojchinov G. Carbonic anhydrase metabolism is a key factor in the toxicity of CO2 and COS but not CS2 toward the flour beetle *Tribolium castaneum* [Coleoptera: Tenebrionidae]. Comp Bioch Physiol Part C. Toxicol Pharmacol. (2005) 140(1):139–47. doi: 10.1016/J.CCA.2005.01.012 15792633

[78] Domigan LJ Andersson M Alberti KA Chesler M Xu Q Johansson J. Kaplan DL Carbonic anhydrase generates a pH gradient in *Bombyx mori* silk glands. Insect Biochem Mol Biol. (2015) 65:100–6. doi: 10.1016/J.IBMB.2015.09.001 PMC4628561 26365738

[79] Wu G Liu J Li M Xiao Y Yi Y. Prior infection of *Galleria mellonella* with sublethal dose of Bt elicits immune priming responses but incurs metabolic changes. J Insect Physiol. (2022) 139:104401. doi: 10.1016/J.JINSPHYS.2022.104401 35636486

[80] Keehnen NLP Kučerová L Nylin S Theopold U Wheat CW. Physiological tradeoffs of immune response differs by infection type in Pieris napi. Front Physiol. (2021) 11:576797/FULL. doi: 10.3389/FPHYS.2020.576797/FULL 33519499 PMC7838647

[81] Norris K Hopes T Aspden JL. Ribosome heterogeneity and specialization in development. Wiley Interdiscip Reviews: RNA. (2021) 12(4):e1644. doi: 10.1002/WRNA.1644 33565275 PMC8647923

[82] Rodríguez-Almonacid CC Kellogg MK Karamyshev AL Karamysheva ZN. Ribosome specialization in Protozoa parasites. Internat J Mol Sci. (2023) 24(8):7484. doi: 10.3390/IJMS24087484 PMC10138883 37108644

[83] Wojda I Kowalski P Jakubowicz T. Humoral immune response of *Galleria mellonella* larvae after infection by *Beauveria bassiana* under optimal and heat-shock conditions. J Insect Physiol. (2009) 55(6):525–31. doi: 10.1016/j.jinsphys.2009.01.014 19232408

[84] Cytrynska M Zdybicka-Barabas A Jablonski P Jakubowicz T. Detection of antibacterial polypeptide activity *in situ* after sodium dodecyl sulfate-polyacrylamide gel electrophoresis. Anal Biochem. (2001) 299(2):274–6. doi: 10.1006/abio.2001.5422 11730357

[85] Zdybicka-Barabas A Januszanis B Mak P Cytryńska M. An atomic force microscopy study of Galleria mellonella apolipophorin III effect on bacteria. Biochim Biophys Acta (BBA) - Biomembranes. (2011) 1808(7):1896–906. doi: 10.1016/J.BBAMEM.2011.03.013 21453676

[86] Zdybicka-Barabas A Stączek S Pawlikowska-Pawlęga B Mak P Luchowski R Skrzypiec K. Studies on the interactions of neutral *Galleria mellonella* cecropin D with living bacterial cells. Amino Acids. (2019) 51(2):175–91. doi: 10.1007/S00726-018-2641-4/FIGURES/9 30167962

