# Supplementary material for: Immune priming modulates Galleria mellonella and Pseudomonas entomophila interaction. Antimicrobial properties of Kazal peptide Pr13a
Source: Front Immunol. 2024 Feb 26;15:1358247. doi: 10.3389/fimmu.2024.1358247 (PMC10925678; doi:10.3389/fimmu.2024.1358247)
Supplement: Supplementary file 1 [file DataSheet_1.zip › Supplementary Figure S3.DOCX]

Supplementary Figure S3

**Main information concerning identified, on the basis of N-terminal sequence provided in Fig. 6 and NCBI database, Kazal peptide Pr13a**

**Protein:** XP_026749039.1 , no conserved domains (including Kazal domains)

**Name**: Kazal peptide Pr13a

**Gene**: LOC113509813

**Amino acid sequence**:

*MKAFSLFVFAVLLVAVSCRP*DKTDLKQLKAEAARKKACMQDCTSVKVEPLCAGKNGAKSLSFGSECVLHNYNCEHKENFKMISNGQCPGSDGIRLS (96 amino acids). Underlined is a sequence obtained by Edman degradation while the putative propeptide is italicized.

**Calculated molecular weight**: 10 313 Da

**Similarity of the sequence to other proteins**:

| Name/source | Characteristics |
| --- | --- |
| Uncharacterized protein LOC113498246, *Trichoplusia ni*  Hypothetical protein RR46_13217, Papilio Xuthus  Vasotab-like *Hyposmocoma kahamanoa XP_026319014.1*  Uncharacterized protein LOC106122268 *Papilio Xuthus*  Vasotab *Achroia grisella*  Uncharacterized protein LOC120630022 isoform X1 Pararge aegeria  Uncharacterized protein LOC123870219, *Maniola jurtina* | 77% identity  Kazal type serine protease inhibitors and follistatin-like domains, 10 342 Da  76% identity  Kazal type serine protease inhibitors and follistatin-like domains  77% identity,  Kazal type serine protease inhibitors and follistatin-like domains,  Calculated molecular weight 10 339  75% identity  Kazal type serine protease inhibitors and follistatin-like domains  Calculated molecular weight 8 572 Da  91% identity  No conserved domains,  Calculated molecular weight 10 041 Da  74% identity  Kazal type serine protease inhibitors and follistatin-like domains  Calculated molcular weight 10 283 Da  74% identity,  Kazal type serine protease inhibitors and follistatin-like domains,  Calculated molecular weight 10 288 Da |
