# Supplementary figures and images for: Immune priming modulates Galleria mellonella and Pseudomonas entomophila interaction. Antimicrobial properties of Kazal peptide Pr13a
Source: Front Immunol. 2024 Feb 26;15:1358247. doi: 10.3389/fimmu.2024.1358247 (PMC10925678; doi:10.3389/fimmu.2024.1358247)

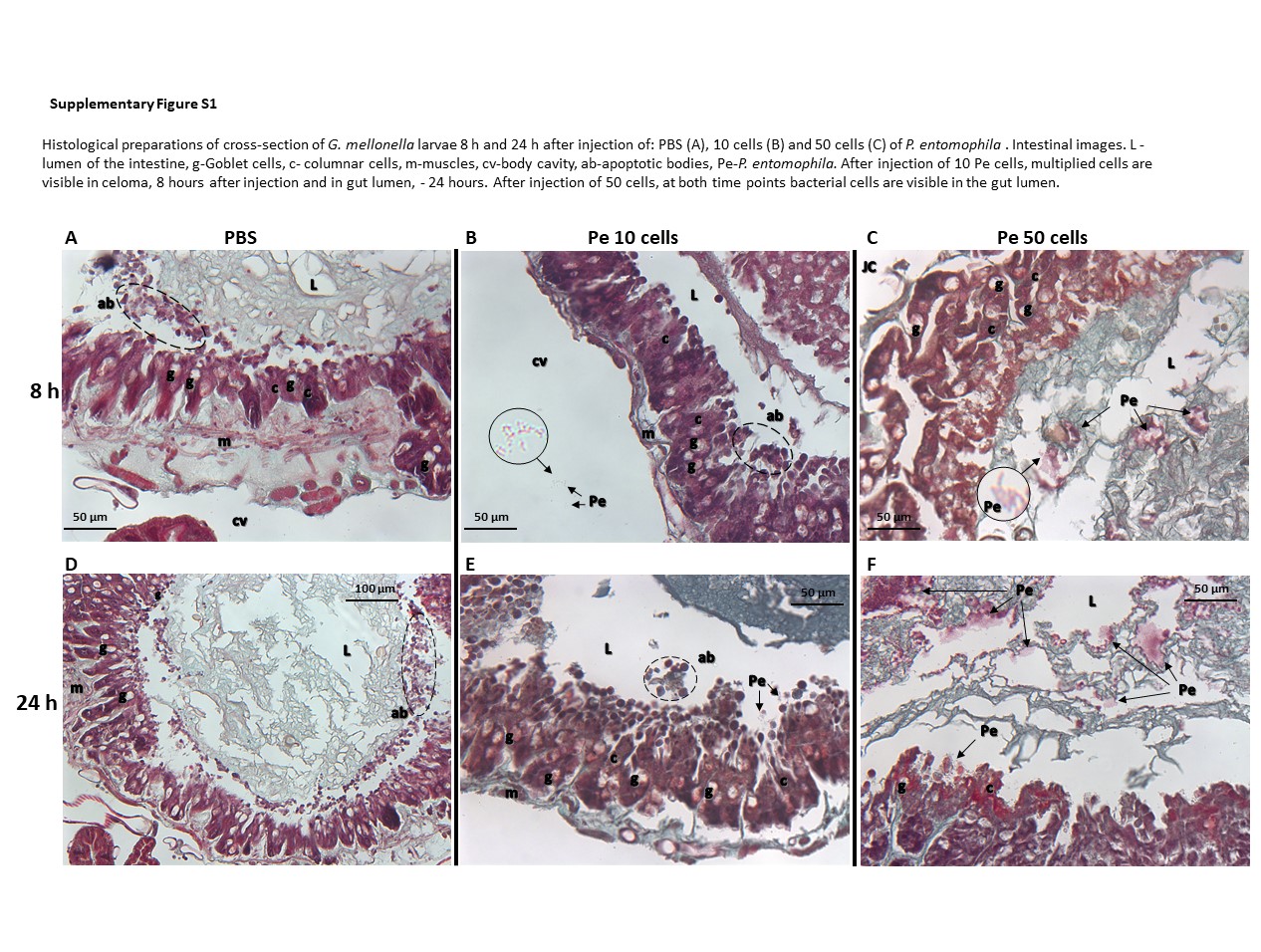

Supplement: Supplementary file 1 [file DataSheet_1.zip › Supplementary Figure S1.JPEG]
